# Public attitudes on performance for algorithmic and human decision-makers

**DOI:** 10.1093/pnasnexus/pgae520

**Published:** 2024-12-10

**Authors:** Kirk Bansak, Elisabeth Paulson

**Affiliations:** Department of Political Science, University of California, 210 Social Sciences Building, Berkeley, CA 94720, USA; Technology and Operations Management Unit, Harvard Business School, Soldiers Field, Boston, MA 02163, USA

**Keywords:** public opinion, algorithmic decision-making, conjoint experiments

## Abstract

This study explores public preferences for algorithmic and human decision-makers (DMs) in high-stakes contexts, how these preferences are shaped by performance metrics, and whether public evaluations of performance differ depending on the type of DM. Leveraging a conjoint experimental design, approximately 9,000 respondents chose between pairs of DM profiles in two high-stakes scenarios: pretrial release decisions and bank loan approvals. The profiles varied by type (human vs. algorithm) and three metrics—defendant crime rate/loan default rate, false positive rate (FPR) among white defendants/applicants, and FPR among minority defendants/applicants—as well as an implicit fairness metric defined by the absolute difference between the two FPRs. The results show that efficiency was the most important performance metric in the respondents’ evaluation of DMs, while fairness was the least prioritized. This finding is robust across both scenarios, key subgroups of respondents (e.g. by race and political party), and across the DM type under evaluation. Additionally, even when controlling for performance, we find an average preference for human DMs over algorithmic ones, though this preference varied significantly across respondents. Overall, these findings show that while respondents differ in their preferences over DM type, they are generally consistent in the performance metrics they desire.

**Significance Statement**

Algorithmic decision-making tools are increasingly adopted in high-stakes domains like criminal justice, healthcare, and finance. Recently, media coverage has exposed the public to issues of bias, (in)accuracy, and (un)fairness in algorithms, sparking public outcry and legislative action. Yet, little is known about which attributes the public values in algorithmic decision-makers (DMs), how they would prefer to trade-off attributes such as efficiency and fairness, and whether these preferences vary by DM type (algorithmic vs. human). Using a large-scale conjoint experiment, this study finds that while the public holds conflicting views about their preferred type of DM, they have largely consistent preferences with respect to performance metrics, prioritizing efficiency over fairness regardless of DM type.

## Introduction

Concerns about the efficacy, bias, and fairness of AI and algorithmic decision-making are not new, but they have intensified as algorithmic decision-making has increasingly diffused into public policy and other high stakes decision-making contexts, such as the criminal justice (1) and healthcare (2) systems. Recent research has provided mixed evidence on the effectiveness and fairness of algorithmic decision-making in different realms (e.g. (1, 3–6)). Through the dissemination of academic research along with media coverage, the public has become increasingly aware of both the potential benefits and risks of the use of algorithms in the public sphere.

In particular, several high-profile cases in recent years have spotlighted failure points in the performance of algorithmic decision-making systems—perhaps most notably related to unfairness and/or bias towards certain groups. For example, in 2016, the news outlet ProPublica published an article titled “Machine Bias” that analyzed an algorithm used to generate risk scores for criminal defendants, ultimately arguing that the algorithm was biased against Black defendants (7). This article garnered massive interest and debate—in both the academic and public sphere—over algorithmic bias and definitions of fairness (8–10). In another high-profile example, a 2019 article in *Science* highlighted evidence of racial bias in an algorithm being used by a major US hospital system to allocate specialized healthcare resources to patients (6), with hundreds of news outlets disseminating the findings of this study to the public.^a^ As a final example, consider the ongoing public discourse surrounding the use of algorithms in mortgage loan decisions. Mainstream news articles have pointed out both the potential benefits of digital/algorithmic loan platforms, arguing that they can result in less biased mortgage loan decisions when compared to human loan officers (11), and the pitfalls of traditional algorithmic credit assessments, arguing that these algorithms lead lenders to reject Black applicants with much higher likelihood than similar white applicants (12).

Debates over such algorithmic tools have not only become frequent in mainstream media but also spurred public-facing legislative and governmental activities. In a 2020 statewide referendum, Californians voted against a plan to replace cash bail with algorithmic risk assessments. This was a surprising result for much of the state’s political elite, given California’s status as a progressive state where cash bail has long been an unpopular practice. The electorate’s decision came after a roughly $10 million opposition campaign that portrayed algorithmic risk assessments as discriminatory. Indeed, in the state’s official voter guide, the lead argument presented by the opposition emphasized these considerations of unfairness and algorithmic bias against minorities and the poor (13). In addition, elected officials have initiated and publicized a number of high-level governmental efforts (at federal, state, and local levels) aimed at achieving more responsible, effective, and equitable use of algorithmic decision-making and AI in society. This includes the Biden Administration’s 2023 Executive Order on the Safe, Secure, and Trustworthy Development and Use of Artificial Intelligence (14); 2024 legislation in Washington state that created an Artificial Intelligence Task Force (15); and New York City Mayor Bill de Blasio’s 2018 creation of an Automated Decision Systems Task Force in accordance with a legislative mandate by the New York City Council (16).

In all of these examples—including media, legislative, and governmental activities—the public has increasingly learned about concerns related to specific features of algorithms (e.g. their potential for bias) and has been steadily pulled into debates about algorithmic decision-making. Such developments underscore the theoretical and policy importance of better understanding public attitudes and expectations regarding algorithmic decision-makers (DMs). There are two bodies of relevant scholarship in this area. First, there has been a limited amount of research focused on how the public (as *observers and voters*) views the use of AI and algorithmic decision-making tools in society. Past surveys of the public have found negative views on AI and institutions managing the use of AI to be pervasive (17–21). Other studies have highlighted that public trust in algorithmic DMs is relatively malleable (22), and contingent upon the types of task being performed (e.g. whether the task is more “mechanical” or “human” in nature, or the degree to which it involves assisting or sanctioning people) (23, 24) and various design features (18, 25).

Second, a larger body of research has focused on the views and behaviors of human *users* of AI and algorithmic decision-making tools. The reluctance of human users to rely on algorithmic advice—a phenomenon termed “algorithm aversion”—has been well-documented by a growing body of research (e.g. (26, 27)). At the same time, “algorithm appreciation”—a phenomenon whereby humans prefer to rely on algorithmic advice than human advice—has also been uncovered in certain settings (28). This body of work has found the degree of aversion to (or appreciation for) algorithmic decision-making among users to be context-dependent (28–34). For example, algorithm aversion may be mitigated, though not erased, by providing users with performance metrics about the algorithmic DM (e.g. related to accuracy, fairness, etc.) (29, 34–36). On the other hand, if the algorithm’s performance is worse than expected, this can increase algorithm aversion and decrease appreciation (28, 37).

However, many open questions remain about public attitudes toward performance metrics, preferences for tradeoffs between them, and how DM type (algorithm vs. human) influences these views. Broadly speaking, it is unclear to what degree public attitudes toward algorithmic DMs in society can be shaped through transparency about different performance metrics. Furthermore, it is also unclear how the public considers the performance metrics relative to one another. While it is well-known among experts that simultaneously maximizing both fairness and accuracy of predictive algorithms can be impossible, leading to unavoidable tradeoffs between these goals (38, 39), such tradeoffs are typically not highlighted in public-facing media coverage and governmental initiatives. Relatedly, it is largely unknown how the public prefers to trade off these various objectives and whether such preferences differ when considering algorithmic vs. human DMs. As may be suggested by the numerous controversies and governmental initiatives highlighting concerns over algorithmic bias and discrimination, the fairness of these tools might be a particularly salient consideration for the public. As such, we might expect not only that the public places a high priority on fairness relative to other performance metrics, but also that such prioritization may be more pronounced when evaluating algorithmic DMs than human DMs.

The purpose of this study is to understand how DMs’ performance metrics (efficiency and fairness) influence public evaluations and preferences for human and algorithmic DMs, and whether the importance of these metrics is contingent upon DM type. To perform this investigation, we designed, preregistered, and implemented a high-dimensional conjoint experiment on a large opt-in sample (n≈9,000) constructed to be representative of the US population. In this experiment, respondents were asked to evaluate pairs of competing DMs (more details below). Conjoint experimentation is a survey-experimental technique in which respondents are asked to indicate preferences over, or rate, alternatives that vary across multiple attributes, where the alternatives are typically presented in the form of a table (for an overview, see Ref. (40)). Randomly varying multiple attributes at the same time allows for estimation of the effects of the attributes and comparison of their relative influence. Researchers have also argued that eliciting preferences and attitudes via conjoint experiments can reduce social desirability bias and recover more valid measures of beliefs in complex and multidimensional settings (41–43), an important consideration in research focused on multifaceted and potentially controversial topics like those considered in this study.

To the best of our knowledge, this is the first extensive study to control for both DM type (human vs. algorithmic) *and* multiple performance metrics (while allowing the performance metrics to vary across instances). Furthermore, in contrast to previous studies that have manipulated performance metrics, this study takes a high-dimensional approach to doing so by employing quasi-continuous randomized features. This design allows for a clearer understanding of the public’s relative sensitivities with respect to multiple competing performance priorities, how the public is willing to trade off between different performance metrics including fairness, and whether this varies by DM type.

## Summary of research design

In our study, each respondent was randomly presented with one of two high-stakes scenarios, which would form the context for the conjoint experiment: (i) a criminal defendant pretrial release scenario, where the central issue was whether defendants should be held in jail or released prior to their trial (“Crime scenario”); or (ii) a bank loan scenario, where the central issue was whether applicants should be given or denied loans (“Loans scenario”). These scenarios both represent prominent algorithmic decision-making contexts in public discourse, and we opted to include two different scenarios in our design to assess the robustness of any results. While both are high-stakes decisions and involve judgment-based (rather than mechanical) determinations, these domains have deep substantive differences. For example, bank loans are the decisions of private companies whereas pretrial release decisions are made by public institutions. In addition, bank loan and pretrial release decisions have fundamentally different impacts on individuals and society. Hence, including these two distinct scenarios gives us the ability to confirm that our findings are not idiosyncratic to one unique scenario, thus giving us greater confidence in their broader generalizability.

The conjoint experiment asked respondents to evaluate pairs of hypothetical DMs, where the DMs were situated within the scenario under consideration. In the Crime scenario, the DMs were responsible for making decisions about pretrial release, and in the Loans scenario the DMs were responsible for granting or denying loans. Furthermore, each respondent was also randomized into one of three conditions regarding the type of DMs in each pair they evaluated: (i) a “Humans condition” where both DMs were human judges or bank managers, (ii) an “Algorithms condition” where both DMs were algorithms, and (iii) a “Faceoff condition” where the pair included one human and one algorithmic DM.

**Fig. 1.**
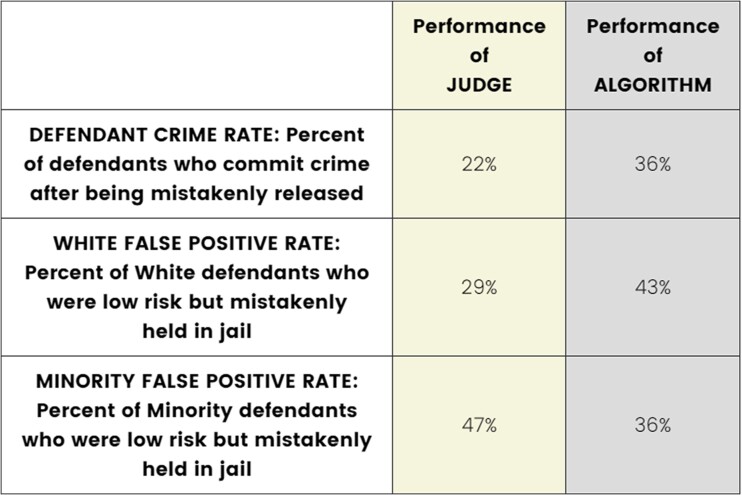
Example of conjoint profile pair: Crime scenario, Faceoff condition.

In addition to providing information on the DM type, each DM had a profile that varied on performance metrics. Specifically, we independently and simultaneously varied information about three performance attributes: (i) the DM’s Defendant Crime Rate (percent of defendants who commit a crime after being mistakenly released by the DM) or the Loan Default Rate (percent of applicants who default on loans that have been granted by the DM), (ii) the DM’s white false positive rate (WFPR, percent of White defendants/applicants who were mistakenly held in jail/denied a loan), and (iii) the DM’s minority false positive rate (MFPR, same but for minority defendants/applicants). The values for the performance metrics were drawn from a wide range of predetermined integer values, making for effectively continuous attributes and ultimately allowing for the estimation of more flexible response surfaces than is normally possible in conjoint analysis (for another example of conjoint research employing quasi-continuous attributes, see Ref. (44)). Figure 1 provides a randomized example of what respondents were shown, in this case in the Faceoff condition of the Crime scenario. Additional examples are provided in Figs. S1–S3.

Collectively, the three attributes capture the degree to which the DM’s decisions are (in)efficient, in the sense that they correspond to undesirable costs: The crime/default rate represents costs to society, and the false positive rates (FPRs) each represent costs to individuals (the DM’s subjects).

In addition, the absolute difference between the two FPRs captures the degree to which the DM’s decisions are (un)fair, corresponding to a definition of (un)fairness in decisions across groups that is well-established in the algorithmic fairness literature (45–47). While other definitions of (un)fairness exist, this definition is relatively intuitive and easy to communicate, and it has become particularly prominent in applied settings. On top of various influential studies having focused on this definition (e.g. (4, 7, 39)), it has become the operationally salient fairness consideration in practice in various areas. For instance, algorithmic auditing systems have prioritized the consideration of equal FPRs (e.g. (48)), and the National Institute of Justice’s (NIJ’s) “Recidivism Forecasting Challenge” employed the difference in FPRs as its sole criterion for evaluating fairness (49).

The survey text provided explicit language to highlight equality of FPRs as a salient performance metric in addition to the others. Specifically, in introducing the scenarios prior to the experiment, the survey text described how mistakes by the DMs could result in three specific problems: costs to society in the form of crime or unpaid loans, costs to individuals in the form of “false positive” decisions that wrongly hold individuals in jail or deny them loans, and racial disparities in the form of unequal FPRs across racial groups. With respect to the latter consideration, the survey text explained how differences in FPRs raise “concerns about fairness and race-based discrimination.” As such, the survey instrument explicitly highlighted fairness and framed this fairness metric as one of the core elements of DM performance, along with the other metrics. At the same time, respondents were not visually guided to prioritize fairness in their evaluations; that is, while respondents could directly deduce the difference in the White and Minority FPRs displayed in each profile, the difference itself was not calculated and displayed for the respondent. This design decision was made in order to understand the extent to which respondents would naturally incorporate these fairness considerations into their evaluations and weigh fairness against different efficiency considerations, without visually priming them to do so.

Each respondent evaluated 10 pairs of DMs. For each pair, respondents were asked to (i) choose their preferred DM in the pair (the “choice” outcome) and (ii) individually rate the performance of each DM on a scale from 1 (very bad) to 7 (very good) (the “rating” outcome). Hence, we are able to understand how people’s preferences/attitudes regarding DMs varies jointly with DM type (human vs. algorithm), efficiency, and fairness in the context of two high-stakes policy scenarios. In addition, we also included a number of direct questions asking respondents about their expectations regarding the performance of different DM types and their performance priorities.

For more details on the sample, survey text, and experimental design, see the Materials and methods section.

## Results

### Relative importance of performance metrics

In this section, we investigate the degree to which people’s preferences are sensitive to different performance metrics. Table 1 shows the results of a linear regression of the choice outcome on the four performance metrics using data from all conditions. Recall that the survey text specifically defined fairness as the (absolute) difference between MFPR and WFPR. Thus, when analyzing fairness as a performance metric, it is constructed in this same way. Because fairness *decreases* as the absolute difference in FPRs increases, we refer to this metric as *unfairness* in tables and figures. Also note that while unfairness is constructed from WFPR and MFPR, it is not correlated with either and is therefore valid to include in a linear regression along with MFPR and WFPR. A regression using only unfairness and crime/default rate is also included in the Table S15, and the coefficients on these variables remained virtually unchanged in that specification.

**Table 1.** Linear regression results predicting respondent choice using data across all conditions.

	Crime scenario	Loans scenario
Crime/Default rate	− 0.011*** (0.0002)	− 0.043*** (0.001)
WFPR	− 0.006*** (0.0002)	− 0.026*** (0.001)
MFPR	− 0.008*** (0.0002)	− 0.036*** (0.001)
Unfairness	− 0.002*** (0.0002)	− 0.008*** (0.001)
Constant	1.275*** (0.010)	1.049*** (0.006)
Observations	95,680	84,800
$R^{2}$	0.127	0.138

n=95,680 (4,784 respondents each evaluating 20 DM profiles) in Crime scenario, and n=84,800 (4,240 respondents) in Loans scenario. Standard errors shown in parentheses. Note: Our preregistration plan focused on estimating these results separately by condition (see Tables S11–S14); however, given limited heterogeneity across condition (discussed later), we provide this succinct presentation of results here.

* P<0.1; ** P<0.05; *** P<0.01.

As shown in Table 1, the crime/default rate has the strongest effect on respondent choice, followed by MFPR, WFPR, and unfairness. This ordering is consistent across scenarios. Overall, Table 1 indicates a high willingness to trade off fairness for improvements in the other three metrics. Consider the following concrete example: In the Crime scenario, on average respondents place the same value on decreasing unfairness from a twenty percentage-point difference in FPRs to zero—i.e. eliminating half of the maximum possible difference—as they place on decreasing both FPRs by only three percentage points (or decreasing just one FPR by approximately five to seven percentage points). Respondents also make similarly large tradeoffs between fairness and the crime/default rate, though the crime/default rate is not necessarily directly comparable to fairness.

While Table 1 reports the effects when imposing a linear specification, we confirm that these estimates are informative by verifying that the marginal response surfaces estimated via LOESS are indeed roughly linear (Fig. S4). In addition, we also calculate the permutation variable importance of each metric using a more flexible polynomial model and find consistent relative importances across the metrics (see Table S10). Relatedly, we also construct isocurves using flexible polynomial models (estimated via ridge regression) to further analyze the tradeoffs respondents are willing to make across the metrics (Figs. S5 and S6).

In addition, we consider the substantive magnitude of the effects of the performance metrics by converting the results in Table 1 into more intuitive quantities. Specifically, we consider increasing each performance metric by various increments (e.g. an increase of 50% of the total range of values taken by the metric) and then compute the corresponding effects on respondent choice. Further, we compute both the raw effects (i.e. the effect on the probability of choice) and the standardized effects (i.e. effect in terms of standard deviations of the outcome). The results are shown and discussed in Tables S2 (Crime scenario) and S3 (Loans scenario). The results suggest that changes in the performance metrics are capable of inducing substantively important influences on respondent choice, with the possible exception of the unfairness metric. The findings are also similar when analyzing the results for the rating outcome variable (see Tables S4 and S5).

Heterogeneous effects based on race, political party, education level, and beliefs about AI are shown in Tables S25–S32, in the columns corresponding to “All data.” While we do see some heterogeneity, the effects are mostly consistent in their relative sizes and do not reveal fundamentally conflicting views on the performance metrics across groups. Some potentially noteworthy differences are that the relative gap between the effects of MFPR and WFPR (with MFPR being prioritized over WFPR) is particularly large for nonwhite respondents and for Democratic respondents, whereas this gap shrinks for white respondents and disappears for Republican respondents.

In addition to analyzing the effects of the performance metrics on respondents’ choices, we also included a direct question that asked respondents to self-report their performance priorities. Interestingly, the distribution of respondents’ self-reported priorities was somewhat inconsistent with the conjoint choice results. When asked directly, 54% (58%) of respondents reported low crime (default) rate to be their top priority in the Crime (Loans) scenario, consistent with the crime/default rate metrics exhibiting the strongest effect in the conjoint analysis (see Table S9). However, the percentage of respondents reporting fairness as their top priority was higher than the percentage reporting low FPR to be their top priority for both scenarios. Specifically, 26% (24%) of respondents reported fairness to be their top priority relative to only 20% (18%) of respondents reporting low FPR to be their top priority, a statistically significant difference at P<0.001 for both scenarios (Table S9). In other words, respondents seemed to over-report their prioritization of fairness relative to the extent to which they incorporated fairness into their actual choices and preferences over DMs.

Furthermore, differences in the relative importance of each performance metric in the conjoint results are strikingly limited even when we consider subsetting respondents by their self-reported performance priorities. The only notable difference across these performance-priority subsets is that the effect magnitude for the crime/default rate varies, but it remains strong for all subsets nonetheless, and the relative ordering of the other three metrics also remains generally stable across the board (Tables S33 and S34, columns 1–3).

### Consideration of DM type

The results above are aggregated across both human and algorithmic DMs. However, respondents may have different views depending on the DM type.

**Fig. 2.**
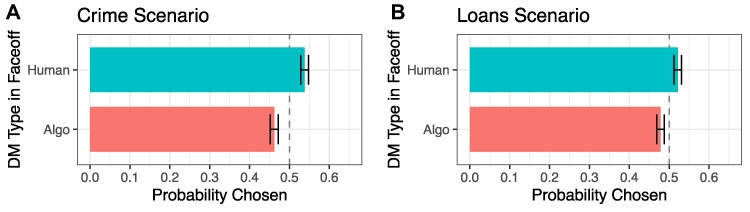
Probability that human or algorithmic DM is chosen in the Faceoff condition in the Crime scenario (A) and Loans scenario (B). n=31,340 (1,567 respondents each evaluating 20 DM profiles) in Crime scenario, and n=28,320 (1,416 respondents) in Loans scenario. 95% CI displayed. Corresponding regression results shown in Table S18.

First, we consider whether there is an average preference for algorithmic or human DMs when they are pitted against each other in the Faceoff condition. Figure 2 shows the probability of each DM type being chosen in the Faceoff condition, revealing an overall average preference for human DMs even though the DMs had equivalent levels of performance by design (i.e. the performance metric values were drawn from the same distributions for both human and algorithmic DMs). Overall, the human DM has a 7.6 percentage-point higher probability of being chosen over the algorithmic DM in the Crime scenario and 4.3 percentage-point higher probability in the Loans scenario (both of which are statistically significant at P<0.0001, see Table S18).

Figure S7 shows similar results when looking at the performance rating outcome variable in which respondents were asked to give their evaluation of the performance of each DM individually. Not only are respondents choosing the human DM more often, but they also rate the human DM’s overall performance in the experiment as stronger (even though, by design, it is not).

**Fig. 3.**
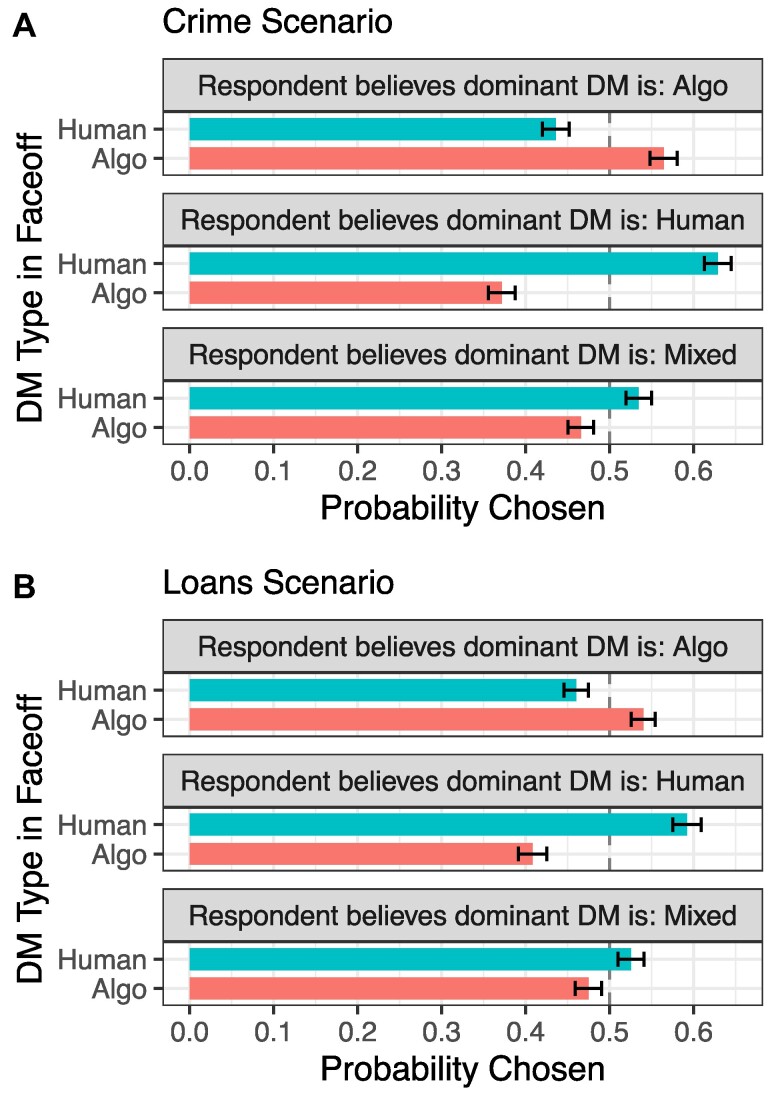
Proportion of time that each DM is chosen in the Faceoff condition, subsetted by respondents’ beliefs about DM performance in the real world. A respondent believes that a certain DM is “dominant” if they believe that it is both fairer and more efficient in the real world. In Crime scenario (A): n=10,200 for Algo dominance (510 respondents that believe Algos are dominant, each evaluating 20 DM profiles), n=11,900 for Human dominance (595 respondents that believe Humans are dominant, each evaluating 20 DM profiles) and n=9,240 for mixed views (462 respondents with mixed views, i.e. a belief that one DM type is more efficient while the other is fairer). In Loans scenario (B): n=10,620 for Algo dominance (531 respondents), n=8,800 for Human dominance (440 respondents), and n=8,900 for mixed dominance (445 respondents). 95% CI displayed. Underlying estimates can be found in Table S8. Note: this analysis was not preregistered.

These average results, however, mask substantial heterogeneity. In particular, there are sizeable subgroups of respondents that favor algorithmic DMs, and those that (even more) strongly favor human DMs. In response to a direct survey question asking the respondents which DM type they believe in the *real world* is (i) more efficient and (ii) fairer (see the Materials and methods for more detail), about 33% (38%) said that algorithmic DMs are both fairer and more effective at keeping crime (loan default) rates low (Table S7). These respondents chose the algorithmic DM 56% (54%) of the time in the Crime (Loan) scenario, and thus exhibit an overall preference for algorithmic DMs, in sharp contrast to the average bias towards human DMs. On the other side, about 40% (30%) expressed a belief that human DMs are fairer and more effective at keeping crime (loan default) rates low (Table S7), and among those respondents the human DM was chosen 63% (59%) of the time (see Fig. 3).

Additionally, though an average preference for human DMs was found across all three political party affiliations (Democratic, Republican, and Independent), the magnitude of the preference varies substantially. On average, those who self-identified as Democrats chose the human DM 4.5 percentage-points more than the algorithmic DM on average in the Crime scenario, whereas this human-DM effect was almost three times larger for Republicans, specifically 12.7 percentage points (see Tables S27 and S28; in the Loans scenario, the magnitude of the effect is roughly twice as large for Republicans than for Democrats). This difference is similar in magnitude to the bias exhibited by those who reported being more pessimistic about the use of AI in society relative to those who reported being more optimistic (see Tables S31 and S32 for results and description of this self-reported measure). Thus, although there is a small average preference for human DMs across the entire pool of respondents, such an effect masks substantial heterogeneity in the data.

**Fig. 4.**
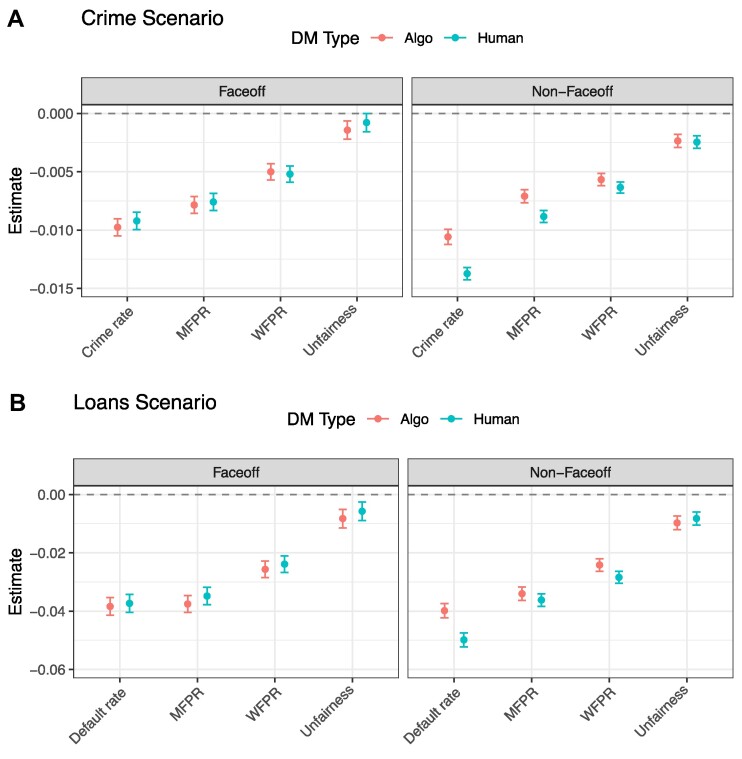
Linear regression coefficients for each condition by DM type. n=31,340 (1,567 respondents each evaluating 20 DM profiles) in Crime scenario (A) Faceoff condition, n=64,340 (3,217 respondents each evaluating 20 DM profiles) in Crime scenario Non-Faceoff conditions, n=28,320 (1,416 respondents) in Loans scenario (B) Faceoff condition, and n=56,480 (2,824 respondents) in Loans scenario Non-Faceoff conditions. 95% CI displayed. Estimates can be reproduced via the numbers reported in Tables S7 (columns 2 and 5) and S19–S20 (column 2).

Second, we consider whether there is heterogeneity in the relative importance of the performance metrics when respondents evaluate human vs. algorithmic DMs. Given that respondents have different preferences for human vs. algorithmic DMs, one might also expect the relative importance of the four performance metrics to vary by DM type. However, our results suggest that DM type has limited bearing upon the importance of the performance metrics, as shown by Fig. 4 (and supported by Tables S17–S22). In the Faceoff condition, the effects are all virtually identical for human and algorithmic DMs, with statistically insignificant differences (Tables S19 and S20). Furthermore, in the two non-Faceoff conditions (Humans condition and Algorithms condition), the effect of unfairness also remains roughly the same (and statistically indistinguishable) for human and algorithmic DMs. In the non-Faceoff conditions, it does appear that the effects of crime/default rate, MFPR, and WFPR are slightly attenuated when algorithmic DMs are being evaluated. Nonetheless, the general structure of the preferences and relative orderings of the effects remain highly robust. We see the same patterns when considering the permutation variable importances (Table S10), and when looking at the rating outcome (Fig. S8, Tables S23 and S24), confirming that our results are not an artifact of the choice outcome. Additionally, we find no subgroups for which the relative importance of the four performance metrics substantially changes when considering human vs. algorithmic DMs. The largest such shift was observed among nonwhite respondents in the Loans scenario, in which the loan default rate was more salient when evaluating human DMs, whereas the MFPR was more salient when evaluating algorithmic DMs (Fig. S9). However, this change is relatively small and not observed in the Crime scenario.

## Discussion

As described above, our data indicate that the structure of preferences over the various performance metrics does not meaningfully vary for the evaluation of algorithmic vs. human DMs. In evaluating both DM types, respondents appear to be most sensitive to the crime rate or loan default rate (the costs to society) and least sensitive to unfairness among the considered metrics. And while all four metrics are statistically significant determinants of respondent choice, the substantive strength of the effect of unfairness is questionable. We note here, however, that conceptually the crime rate/loan default rate may not be directly comparable to either the FPRs or unfairness. Though crime (loan default) rate corresponds to the overall false negative rate (FNR), labeling it as the crime (loan default) rate in the survey instrument could have amplified its perceived importance. This is in contrast to the FPRs, which were labeled as such. Additionally, the overall FPR is split into the WFPR and MFPR. As a result, an increase in the overall FPR may correspond to an increase in *both* the WFPR and MFPR.

On the other hand, the FPRs and unfairness are on the same scale and hence merit direct comparison. In this regard, in both scenarios we see similar evidence of the relative importance respondents placed on considerations of justice vs. fairness, a subtle distinction often drawn in ethics and political philosophy (50). In the scenarios, the individual FPRs represent the degree of *unjust* treatment each group is subjected to; in contrast, the difference in FPRs represents the degree of *unfair* treatment across groups. In both scenarios, respondents on average placed more weight on MFPR than WFPR, indicating an overall belief that unjustly incarcerating a minority defendant or denying a loan to a minority application bears a higher cost than for a white defendant/applicant. The evidence also suggests that the costs resulting from unjust decisions were more of a concern to respondents than unfairness.

Interestingly, the results from the direct question would seem to imply that fairness is a higher public priority than low FPRs, contrary to the actual choices made in the conjoint experiment. In other words, respondents over-reported their prioritization of fairness relative to the extent to which they incorporated fairness into their actual choices. There are at least two possibilities that could explain this discrepancy. First, it may simply reflect greater support for fairness in principle than in practice. Second, the discrepancy could be explained by social desirability bias if respondents felt it was socially expected for them to provide an endorsement of fairness (regardless of the degree to which they actually act upon fairness considerations). Indeed, previous research has highlighted the potential for conjoint experiments to mitigate social desirability bias, relative to other survey methods like direct questions (41–43).

**Fig. 5.**
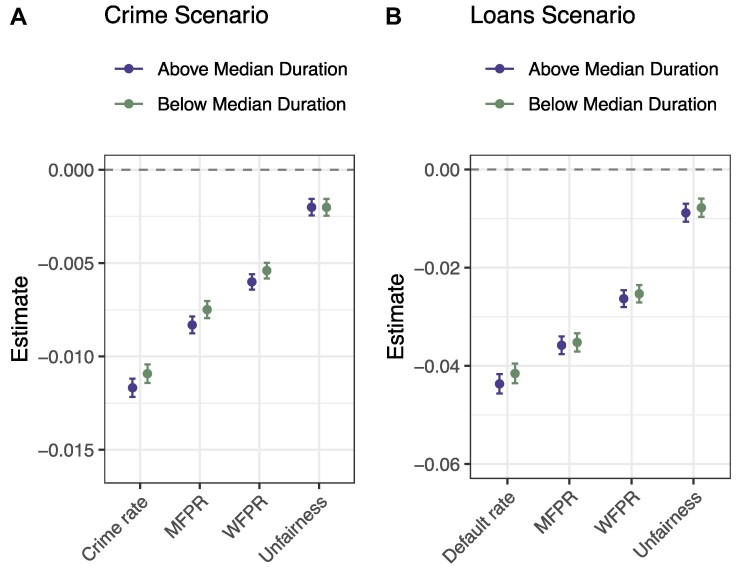
Estimated effects of performance metrics on respondent choice, subsetted by respondents’ time taken on survey (above and below median duration), pooling across all conditions. n=95,680 (4,784 respondents) in Crime scenario (A), and n=84,800 (4,240 respondents) in Loans scenario (B). Note: This analysis was not preregistered.

**Fig. 6.**
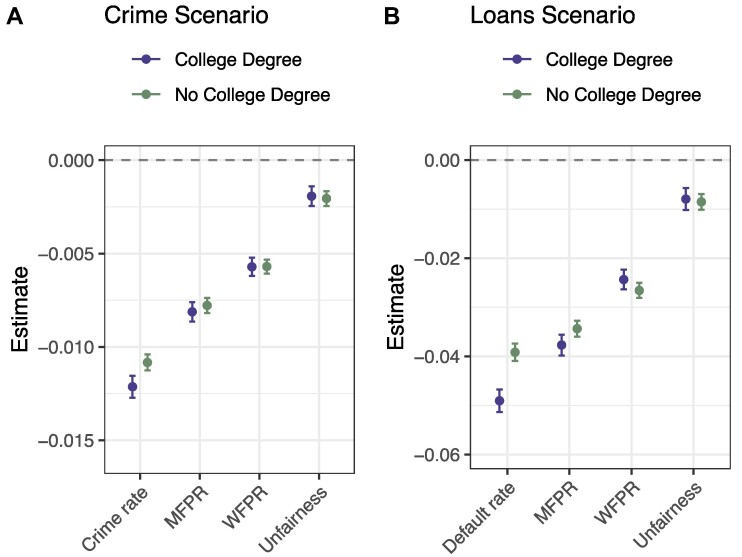
Estimated effects of performance metrics on respondent choice, subsetted by respondents’ education (college degree or no college degree), pooling across all conditions. n=95,680 (4,784 respondents) in Crime scenario (A), and n=84,800 (4,240 respondents) in Loans scenario (B). Note: This analysis was not preregistered.

The limited influence of fairness in guiding the respondents’ choices, along with the fact that this influence does not change depending upon DM type, may be unexpected given the widespread discussions on algorithmic fairness in both public and academic spheres. (Such discussions would seem to suggest a strong emphasis on fairness in particular for algorithmic DMs, though we note that some previous research has actually found there to be less moral outrage sparked by discriminatory behavior by algorithms than by humans (51).) Do these results imply that people actually do not prioritize fairness? Although we provided a clear and straightforward definition of unfairness, and respondents could easily discern the level of unfairness by comparing the two FPRs provided in the conjoint profiles, unfairness was not displayed *on its own* in the conjoint tables. As a result, it is possible that the cognitive burden of needing to calculate unfairness led to its effect being attenuated on average due to inattention and/or satisficing among some respondents. To investigate this possibility, we re-estimate the effects of the performance metrics after subsetting across two dimensions that arguably represent the best proxies for cognitive effort and capacity in our data: respondents’ time spent on the survey and their education level. The results, based on linear regressions that pool the data across all three conditions for each scenario, are shown in Figs. 5 and 6. As can be seen, there are limited differences in the effects of the performance metrics across subsets, and the effects are virtually identical in particular for the unfairness metric. Indeed, none of the differences in the effect of unfairness across subsets are statistically significant.^b^

Notwithstanding the results above, it is still unclear how exactly respondents would behave if fairness were explicitly displayed. Nonetheless, our data at the very least indicate that even with a clear and simple definition, respondents across the board did not actively seek to make fairness a top priority when making decisions: they either did not deem it to be important enough to calculate or did not deem it to be as influential as the FPRs themselves.

The general preference for human DMs over algorithmic DMs detected in the Faceoff condition is consistent with existing literature on algorithmic aversion, though we emphasize that this average effect masks substantial heterogeneity. Specifically, the overall preference for choosing human DMs (and the higher average rating given to human DMs) is predominantly driven by participants who reported a belief that human DMs actually perform better in the real world, and vice versa. We also find that respondents self-identifying as Republicans, or those pessimistic about the use of AI in society, were far more likely to favor the human DM (Tables S27, S28, S31, and S32).

The noteworthy heterogeneity in the bias with respect to DM type leads naturally to questions about whether the relative importance of the four performance metrics is also dependent on DM type. As noted earlier, given the emphasis on fairness in various high-profile controversies covered by the media as well as the stated priorities of recently created governmental task forces on AI, one might assume that the public cares more about fairness when evaluating algorithmic DMs relative to human DMs. Yet, we did not find evidence that the public fundamentally changes their preferences over the four performance metrics based on DM type. It is theoretically possible, however, that this consistency in the relative importance of the metrics across DM type only holds on average, and that certain subgroups do exhibit differences in their relative ranking of the performance metrics when evaluating each DM type. For example, one might hypothesize that those who are pessimistic about the use of AI in society would care more about fairness when evaluating algorithmic DMs. However, our results show that the importance placed on the four metrics is largely consistent across both DM types *and* subsets of the population. While there are some small deviations from the general pattern, the relative ordering of the importance of the four metrics did not dramatically differ for any of the subgroups we analyzed when evaluating human vs. algorithmic DMs (Figs. S10–S14). Given the substantial heterogeneity in preferences over DM type, resulting in the existence of sizeable subgroups with fundamentally opposing views, the limited heterogeneity in the relative importance of the four performance metrics is striking.

**Fig. 7.**
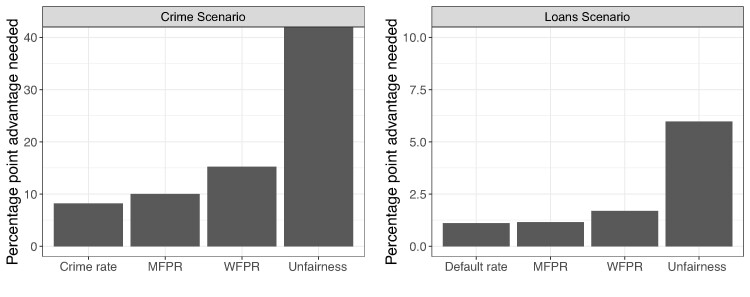
Average percentage point decrease in each metric needed for algorithmic DM to have the same probability as being chosen as the human DM, using Faceoff data. n=31,340 (1,567 respondents each evaluating 20 DM profiles) in Crime scenario, and n=28,320 (1,416 respondents) in Loans scenario. See Table S18 for corresponding regression results.

As a final summary of our results, we can consider the preferences for DM type in concert with the effects of the performance metrics to see how superior performance can counteract preferences for DM type. To illustrate, Fig. 7 measures the performance advantages in terms of each metric that would enable algorithmic DMs to have an equal chance of being chosen as human DMs on average in the Faceoff condition. For example, in the Crime Scenario (Fig. 7 left), an algorithmic DM producing a crime rate that is 8 (or more) percentage points lower than an otherwise identical human DM would be chosen by a majority. As the value of the Unfairness bar exceeds 40 percentage points (the maximum value) for the Crime Scenario, this indicates that there is no fairness level that, with other metrics being equal, would lead to an average preference for an algorithmic DM over a human DM. Figure S15 shows the same results separately for Democrats and Republicans. Though the *absolute* magnitude of the bars is larger for Republicans (indicating a stronger preference for human DMs) the *relative* magnitude of the bars is roughly consistent (indicating similar relative importance placed on each metric). The same patterns hold true for many subgroups considered. Namely, different subgroups can have varying preferences over DM type but are largely consistent in their preferences for DM performance.

### Implications

The results in this study have implications for how algorithmic decision-making systems are designed and conveyed in society, as well as for garnering public support for better-performing DMs, whether they be human or algorithmic. Specifically, these insights highlight what may be some of the more straightforward vs. more challenging considerations in deploying algorithmic DM tools or advocating for superior DMs (human or algorithmic) in high-stakes contexts.

To begin, these tasks are made more straightforward by the strength of the collective influence of the performance metrics relative to any underlying bias against any DM type. Recall that although there is a general inclination towards human DMs even when accounting for performance, this average effect of DM type is small to moderate, and it is driven by only a subset of the population. Furthermore, the influence of performance metrics collectively appears to outweigh the importance of DM type itself. These results indicate that any bias or aversion against algorithmic (or human) DMs is relatively contained and can be eclipsed by performance considerations.

Additionally, the relative importance of the performance metrics is largely consistent across DM type and across population subsets. The consistency across DM type indicates that it is not necessary to formulate distinct arguments, tap into unique psychological constructs, or appeal to varying forms of logic when advocating for different DM types. For instance, there does not appear to be a need to deal with fairness concerns that are unique to AI and algorithmic decision-making.

This is buttressed by the general consistency in respondents’ preferences across population subsets. Even across socially and politically salient cleavages in society—such as race and party identification—any differences in those preferences are fairly limited, rather than fundamentally dissimilar as can occur in some policy realms. This indicates that policymakers and other stakeholders need not deal with the challenge of trying to appeal to disparate (and conflicting) sets of preferences.

Our findings also, however, highlight several key challenges in deploying and communicating about algorithmic (or human) DMs. Most directly relevant to our results is the need to connect fairness concerns—and concerns about any performance metric more broadly—to the existence of tradeoffs. As explicitly indicated by the respondents’ answers to the direct question on performance priorities (with a sizeable percentage naming fairness as their top priority), as well as indirectly suggested by the prevalence of controversies and efforts focused on counteracting algorithmic bias, there seems to be an important predilection for algorithmic fairness (and fairness in general) in society. Yet when induced to consider such issues in the context of tradeoffs as in our conjoint experiments, fairness appeared to be less emphasized than the conventional wisdom may have us believe. This highlights the importance of how competing performance metrics are considered in society by both policymakers and the public—not simply the metrics in isolation but also their relationships and the tradeoffs between them—which merits further research.

Finally, there are various related challenges that are undoubtedly critical but that we do not consider in our study. To begin, even if it is the case that the public’s preferences for fairness are limited on average compared to other metrics, policymakers still need to carefully consider the fairness of decision-making systems that are deployed, insofar as this has important normative and potentially legal implications (e.g. related to equity in society). As a separate issue, even if advocating for a superior DM (whether algorithmic or human) may prove to be straightforward given compelling evidence of its performance advantages, one first needs to generate that evidence and establish its credibility, all of which is no easy task. Furthermore, other issues related to algorithmic DMs that we did not consider in this study (e.g. due process and data privacy issues) could also come into play in debates over the desirability of different DM types.

### Limitations

Our study has several limitations. First, we only consider two distinct scenarios (the Crime scenario and Loans scenario). That being said, both are socially very different scenarios within the scope of high-stakes policy settings that is of interest to us (in contrast to mechanical decision-making tasks), and our results are consistent across both scenarios.

Second, we only consider one definition of fairness, the absolute difference between the group false positive rates. As discussed in more detail in the Introduction, this fairness metric was highlighted to the participants because of its intuitiveness (the idea of false positives and the desirability of equal false positives across groups is easy to communicate), its established social and political salience (the problem of unequal false positive rates is a key fairness concern that has been a focus in media coverage, academic research, and policy settings), and its salience to the fairness-efficiency tradeoff (previous studies have focused on this specific tradeoff). Furthermore, it is unlikely that respondents would generally be aware of the existence of (and conflict between) a multitude of competing fairness metrics. Hence for the purposes of this study, we argue that focusing on unequal false positive rates is appropriate and sufficient for establishing a fairness dimension in the presented decision-making contexts for lay respondents.

Third, the scenarios we presented are stylized, with DMs being either entirely algorithmic or entirely human, rather than presenting hybrid/recommendation systems that are reflective of many real-world implementations. However, we note that our focus here is theoretical: the goal is to understand people’s underlying attitudes, psychology, and belief systems, rather than their view on any singular/concrete implementation. For that reason, we purposefully abstracted away the fine-grained details (which can vary significantly across different contexts and jurisdictions as well) related to how algorithmic tools would be used by or in conjunction with human authorities.

## Materials and methods

### Sample

We fielded our survey in May–July 2022 with 9,030 adult respondents in the United States completing the survey. The sample was an online opt-in sample provided by Qualtrics that was constructed to achieve representativeness with respect to US Census demographic targets on gender, age, region, race/ethnicity, household income, and education. The final sample came very close to exactly meeting the targets; the population targets and sample distributions are displayed in Table S1.

The beginning of the survey included two attention check questions designed to identify respondents who were not seriously engaging with the survey and guard against problems caused by inattentiveness and survey satisficing (52, 53). Both attention check questions asked respondents about content on the same page of the survey; that is, for each question, the correct answer could be found by simply reading the information presented immediately above the question. (More information can be found in the Scenarios section below.) Only respondents who correctly answered both attention check questions were directed to complete the remainder of the survey and comprise our final sample, thus ensuring the representativeness of our final sample with respect to the demographic targets.

### Scenarios

The survey instrument revolved around two scenarios: a criminal defendant pretrial release scenario and bank loan application scenario. Each respondent was randomly presented with only one of the two scenarios.

In the criminal defendant (Crime) scenario, respondents were asked to consider how criminal defendants are treated prior to their trials. Specifically, respondents were told:

On the one hand, criminal defendants can be held in jail while they await their trial. This will protect public safety in case these individuals are dangerous. However, holding defendants in jail is costly for the defendants as well as for the general public, whose tax dollars are used to pay for jail bills. In addition, many defendants will pose no risk to public safety if they are released.

On the other hand, criminal defendants can instead be released from jail while they await their trial. This could be justifiable on the grounds that they have not yet been (and might not be) found guilty of the charged crime, and this could help to save taxpayer dollars. However, certain defendants may commit a crime while awaiting their trial if they are released, further endangering public safety.

Respondents were then given information about how this decision is made on a case-by-case basis in many jurisdictions in the United States, with defendants that are deemed to be “high risk” being held in jail, and defendants deemed to be “low risk” released. The first attention check question asked respondents about what happens to low risk vs. high risk defendants.

In the bank loan (Loans) scenario, respondents were asked to consider decision-making in the consumer banking industry. Specifically, respondents were asked to focus on the key decision point over whether banks decide to provide loans or not to individual applicants, and they were given information about the potential costs involved on both sides (analogous to the specific text shown above for the Crime scenario). Respondents were then given information about how this decision is made on a case-by-case basis, with applicants that are deemed to be “high risk” of defaulting being denied, and applicants deemed to be “low risk” given loans. The first attention check question for respondents in the Loans scenario asked about what happens to low risk vs. high risk applicants.

In both scenarios, the survey text also described how it is unavoidable that occasional mistakes will be made, whereby individuals are incorrectly treated as high or low risk. The survey text highlighted how such mistakes could result in three specific problems: costs to society in the form of crime or unpaid loans, costs to individuals in the form of “false positive” decisions that wrongly hold individuals in jail or deny them loans, and racial disparities in the form of unequal false positive rates across racial groups. With respect to the latter consideration, the survey text explicitly explained how unequal false positive rates is a concern about fairness and race-based discrimination. A second attention check question was used to confirm that respondents took up the information provided to them on what false positives are.

### Experimental design

As described above, subjects were presented with either one of the two scenarios (Crime or Loans), which was randomly assigned. In addition, a set of experiments were embedded within each scenario. Specifically, respondents were presented with a conjoint experiment in which they evaluated DMs within the context of their scenario. In the Crime scenario, this pertained to decisions on whether to hold defendants in jail or release them, and in the Loans scenario this pertained to decisions on whether to give applicants loans or deny them.

In the conjoint experiment, respondents were asked to choose between (as well as individually rate) two DMs in a pair, where the DMs varied in terms of their levels of performance. Specifically, three performance attributes were displayed for each DM. The first was the Defendant Crime Rate (percent of defendants who commit a crime after being mistakenly released) or the Loan Default Rate (percent of applicants who default on their loans) for the DM. The second metric was the White False Positive Rate (percent of White defendants who were low risk but mistakenly held in jail/percent of White applicants who were low risk but mistakenly denied). The third metric was the MFPR (percent of Minority defendants who were low risk but mistakenly held in jail/percent of Minority applicants who were low risk but mistakenly denied). In addition, we also analyzed as a fourth feature the absolute difference between the two false positive rate metrics, which corresponds to the degree of unfairness in the decisions between groups.

For each scenario, the values displayed for each metric were uniformly and independently randomized both within and across all DMs, with values drawn from a predetermined range of integers. For the Crime scenario the range was from 10 to 50, and for the Loans scenario the range was from 0 to 10. In both cases, all integers within the range were possible values, making for quasi-continuous attributes. In addition, another experimental manipulation was layered on top of the conjoint experiment. Specifically, respondents were randomly assigned to one of three conditions: a “Humans” condition where both DMs in the pair were humans (judges/bank managers), an “Algorithms” condition where both DMs were algorithms, and a “Faceoff” condition where the pair included one human and one algorithm. The condition was held constant within-respondent so that, for instance, a respondent in the Faceoff condition would choose between a human and an algorithm for all of the DM pairs they evaluated. Note also that the order in which the human and algorithm were presented in the Faceoff condition was randomized between respondents. The randomization of these conditions allows us to analyze how respondents’ responses/sensitivity to the DMs’ performance attributes is impacted by the DM types (human or algorithm).

The comparisons were presented in the form of tables (examples provided in Figs. S1–S3), and each respondent was presented with 10 comparisons total. Hence, each respondent evaluated a total of 20 different DMs.

Finally, two other randomizations were also implemented: whether the algorithms in question were developed by university researchers or a private firm, and whether the decisions being made take place within the subject’s own locale or within a large generic city. All randomizations were independent and uniform. In our analyses, we pool our results over these two randomizations as they had limited influence on the results.

The full survey instrument, text, and design can be found in our preregistration plan submitted at https://osf.io/twu4h before the start of the survey.

### Outcomes

The primary outcomes of interest relate to the respondents’ evaluations of the DMs presented to them in the conjoint experiment. As described above, respondents were presented with pairs of profiles of DMs. For each pair, respondents were asked to choose the one DM they would prefer making decisions. We use the answers to this question to construct a forced “choice” outcome variable, which is an indicator for whether or not any single DM profile was preferred. In addition, respondents were also asked to provide their evaluation of the performance of each individual DM. Specifically, they were asked the following:

On a scale from 1 to 7, where 1 indicates VERY BAD and 7 indicates VERY GOOD, how would you rate the performance of [each of these DMs]?

We use the answers to this question to construct a performance “rating” outcome variable.

### Direct questions

The survey included several direct questions about respondents’ attitudes toward algorithmic vs. human decision-making.

Respondents were directly asked about their decision-making performance priorities. Specifically, respondents assigned to the [Crime/Loans] scenario were asked: “Which of the following goals do you think is most important in making [decisions on pretrial release/loan decisions]?” and then prompted to rank the following three options:

- Keeping the [criminal defendant crime/loan default] rate low
- Keeping the false positive rate low for [criminal defendants/applicants]
- Keeping the false positive rate equal across White and Minority [criminal defendants/applicants]

This question was posed to respondents directly following the 10 pairwise evaluations.

In addition, respondents were asked about their expectations regarding algorithmic and human DMs in the real world. Following the question above, respondents were told: “Note that the comparisons previously presented to you were purely hypothetical and did not perfectly reflect actual [crime rates/loan default rates] and false positive rates in the real world.” Then, respondents assigned to the [Crime/Loans] scenario were asked two questions. First, “In the real world, which do you think does the best job at keeping [crime/loan default] rates low, [Judges/Managers] or Algorithms?” Second, “In the real world, which do you think does the best job at promoting fairness (i.e. keeping false positive rates equal across White vs. Minority [defendants/applicants]), [Judges/Managers] or Algorithms?” For both questions, respondents were prompted to rank the following three options:

- Judges/Managers
- Algorithms (developed by a university)
- Algorithms (developed by a private firm)

We code respondents as believing algorithms to be the best performer for each respective question if they selected as their top ranked performer either of the algorithm options, “Algorithms (developed by a university)” or “Algorithms (developed by a private firm).”

### Analysis

We analyze the results of our conjoint experiment in several ways. When we evaluate the probability of a certain DM type being chosen, we estimate the marginal means (using the choice outcome for Fig. 2 and the rating outcome for Fig. S7) associated with each DM type in the Faceoff condition. To estimate and perform statistical inference on the difference between the probability of choosing a human vs. algorithm, we use linear least squares regression and regress the outcome on the DM type indicator. Since respondents evaluated multiple conjoint profiles, for all analyses standard errors are clustered by respondent. When we assess the relative importance of the different performance metrics, we estimate the marginal effects of each of the four metrics (default/crime rate, WPFR, MFPR, and unfairness) via linear least squares regression, specifically regressing the choice outcome on the four numerical metrics. These results are shown in Table 1. We further break these results down by condition (Human vs. Human, Alg vs. Alg, and Faceoff), with the results shown graphically in Fig. 4. To estimate and perform statistical inference on the extent to which any effects are different from one another across DM type and/or condition, we employ linear least squares regressions on the appropriate subset(s) of data with the appropriate interaction terms included.

We use the direct survey questions for two purposes. First, we use respondents’ answers to these questions to create subsets for whom we replicate certain analyses. Second, we compute the distributions of the answers to these questions.

All CI displayed are normality-based 95% CI, and all *P*-values reported are based on two-sided t tests.

All analyses except otherwise noted were prespecified in a preregistered analysis plan submitted at https://osf.io/twu4h before the start of the survey.

### Research ethics

Our research was reviewed and approved by the University of California, San Diego’s Institutional Review Board (protocol ID 803639), Stanford University’s Institutional Review Board (protocol ID 65374), and Harvard University’s Institutional Review Board (protocol ID IRB23-0639). At the beginning of the survey, respondents were provided with information about the study and asked to give their consent to participate by clicking the appropriate button. If they did not give their consent, the survey was ended and none of their information was recorded.

## Supplementary Material

pgae520_Supplementary_Data

## Data Availability

Data and replication code are available on Harvard Dataverse: https://doi.org/10.7910/DVN/1QWTHN.

## Notes

a See the Altmetric data associated with this study.

b We assess the statistical significance of the difference in effects across subsets through additional regressions that include an interaction term between the performance metrics and the subsetting variables.

## Preprints

A preprint of a version of this article is posted at https://osf.io/pghmx.
