# Supplementary material for: Public attitudes on performance for algorithmic and human decision-makers
Source: PNAS Nexus. 2024 Dec 10;3(12):pgae520. doi: 10.1093/pnasnexus/pgae520 (PMC11631221; doi:10.1093/pnasnexus/pgae520)
Supplement: pgae520_Supplementary_Data [file pgae520_supplementary_data.pdf]

Supporting Information for

Public attitudes on performance for algorithmic and human decision-makers

Kirk Bansak and Elisabeth Paulson

Kirk Bansak.
E-mail: kbansak@berkeley.edu

This PDF file includes:

Figs. S1 to S15
Tables S1 to S44

Additional Tables

	Population	Sample
**Gender**		
Male:	48%	47%
Female:	52%	52%
Non-binary:	–	1%
**Age**		
Age 18-34:	30%	28%
Age 35-54:	32%	31%
Age 55+:	38%	42%
**Region**		
Northeast:	17%	17%
Midwest:	21%	20%
West:	24%	23%
South:	38%	41%
**Race**		
White:	75%	74%
Black/AA:	13%	14%
Asian or Pacific Islander:	6%	6%
American Ind./Alaskan Native or Other:	6%	6%
**Hispanic Ethnicity**		
Hispanic:	18%	17%
Non-Hispanic:	82%	83%
**Household Income**		
$<$$50K:	$\sim$35%	38%
$50K-100K:	$\sim$35%	32%
$100K+:	$\sim$30%	30%
**Education**		
No college degree:	65%	65%
4 year degree or higher:	35%	35%

Table S1. Sample Demographics

Metric	Increment (% range)	Raw (pp) effect	Standardized effect
Crime rate	25%	-0.113	0.226
Crime rate	50%	-0.226	0.452
Crime rate	75%	-0.339	0.678
Crime rate	100%	-0.452	0.904
WFPR	25%	-0.057	0.114
WFPR	50%	-0.114	0.228
WFPR	75%	-0.171	0.342
WFPR	100%	-0.228	0.456
MFPR	25%	-0.079	0.158
MFPR	50%	-0.158	0.316
MFPR	75%	-0.237	0.474
MFPR	100%	-0.316	0.632
(Un)fairness	25%	-0.020	0.040
(Un)fairness	50%	-0.040	0.080
(Un)fairness	75%	-0.060	0.121
(Un)fairness	100%	-0.080	0.161

Table S2. Effects of increments of performance metrics on respondent choice, Crime scenario. Increments are in terms of the percentage of the full range of the performance metric values in the experimental design. Both raw effects (on probability) and standardized effects (in terms of standard deviations of the outcome) are displayed. Column 1 denotes the performance metric in question, column 2 indicates a specific increment of increase in that metric in terms of the maximum increase possible in the experimental design, column 3 shows the resulting raw effect on respondent choice, and column 4 shows the resulting standardized effect in absolute value. As can be seen, even the highest increase in (un)fairness possible (i.e. 100% of the maximum) yields standardized effects that are below 0.2. To provide context, a widely used conventional set of standards for describing the substantive size of standardized effects sets the value of 0.2 as the benchmark for a "small" effect (see Jacob Cohen, *Statistical Power Analysis for the Behavioral Sciences*, Routledge, 2013). In contrast, the much larger standardized effects that increments of the other three performance metrics are able to generate get closer to the values of 0.5 and even 0.8 that serve as the conventional benchmarks for "medium" and "large" effects.

Metric	Increment (% range)	Raw (pp) effect	Standardized effect
Default rate	25%	-0.106	0.213
Default rate	50%	-0.213	0.426
Default rate	75%	-0.319	0.639
Default rate	100%	-0.426	0.852
WFPR	25%	-0.065	0.129
WFPR	50%	-0.129	0.258
WFPR	75%	-0.194	0.387
WFPR	100%	-0.258	0.516
MFPR	25%	-0.089	0.178
MFPR	50%	-0.178	0.355
MFPR	75%	-0.266	0.533
MFPR	100%	-0.355	0.710
(Un)fairness	25%	-0.021	0.042
(Un)fairness	50%	-0.042	0.083
(Un)fairness	75%	-0.062	0.125
(Un)fairness	100%	-0.083	0.166

Table S3. Effects of increments of performance metrics on respondent choice, Loans scenario. Increments are in terms of the percentage of the full range of the performance metric values in the experimental design. Both raw effects (on probability) and standardized effects (in terms of standard deviations of the outcome) are displayed. Column 1 denotes the performance metric in question, column 2 indicates a specific increment of increase in that metric in terms of the maximum increase possible in the experimental design, column 3 shows the resulting raw effect on respondent choice, and column 4 shows the resulting standardized effect in absolute value. As can be seen, even the highest increase in (un)fairness possible (i.e. 100% of the maximum) yields standardized effects that are below 0.2. To provide context, a widely used conventional set of standards for describing the substantive size of standardized effects sets the value of 0.2 as the benchmark for a "small" effect (see Jacob Cohen, *Statistical Power Analysis for the Behavioral Sciences*, Routledge, 2013). In contrast, the much larger standardized effects that increments of the other three performance metrics are able to generate get closer to the values of 0.5 and even 0.8 that serve as the conventional benchmarks for "medium" and "large" effects.

Metric	Increment (% range)	Raw effect	Standardized effect
Crime rate	25%	-0.255	0.170
Crime rate	50%	-0.510	0.340
Crime rate	75%	-0.765	0.511
Crime rate	100%	-1.020	0.681
WFPR	25%	-0.150	0.100
WFPR	50%	-0.299	0.200
WFPR	75%	-0.449	0.300
WFPR	100%	-0.598	0.399
MFPR	25%	-0.211	0.140
MFPR	50%	-0.421	0.281
MFPR	75%	-0.632	0.421
MFPR	100%	-0.842	0.562
(Un)fairness	25%	-0.084	0.056
(Un)fairness	50%	-0.167	0.112
(Un)fairness	75%	-0.251	0.168
(Un)fairness	100%	-0.335	0.224

Table S4. Effects of increments of performance metrics on rating, Crime scenario. Increments are in terms of the percentage of the full range of the performance metric values in the experimental design. Both raw effects and standardized effects (in terms of standard deviations of the outcome) are displayed. Column 1 denotes the performance metric in question, column 2 indicates a specific increment of increase in that metric in terms of the maximum increase possible in the experimental design, column 3 shows the resulting raw effect on respondent choice, and column 4 shows the resulting standardized effect in absolute value. As can be seen, even the highest increase in (un)fairness possible (i.e. 100% of the maximum) yields standardized effects that are limited to just above 0.2. To provide context, a widely used conventional set of standards for describing the substantive size of standardized effects sets the value of 0.2 as the benchmark for a "small" effect (see Jacob Cohen, *Statistical Power Analysis for the Behavioral Sciences*, Routledge, 2013). In contrast, the much larger standardized effects that increments of the other three performance metrics are able to generate get closer to the values of 0.5 and even 0.8 that serve as the conventional benchmarks for "medium" and "large" effects.

Metric	Increment (% range)	Raw effect	Standardized effect
Default rate	25%	-0.272	0.179
Default rate	50%	-0.543	0.358
Default rate	75%	-0.815	0.536
Default rate	100%	-1.086	0.715
WFPR	25%	-0.164	0.108
WFPR	50%	-0.328	0.216
WFPR	75%	-0.492	0.324
WFPR	100%	-0.656	0.432
MFPR	25%	-0.252	0.166
MFPR	50%	-0.504	0.332
MFPR	75%	-0.757	0.498
MFPR	100%	-1.009	0.664
(Un)fairness	25%	-0.091	0.060
(Un)fairness	50%	-0.182	0.120
(Un)fairness	75%	-0.274	0.180
(Un)fairness	100%	-0.365	0.240

Table S5. Effects of increments of performance metrics on rating, Loans scenario. Increments are in terms of the percentage of the full range of the performance metric values in the experimental design. Both raw effects and standardized effects (in terms of standard deviations of the outcome) are displayed. Column 1 denotes the performance metric in question, column 2 indicates a specific increment of increase in that metric in terms of the maximum increase possible in the experimental design, column 3 shows the resulting raw effect on respondent choice, and column 4 shows the resulting standardized effect in absolute value. As can be seen, even the highest increase in (un)fairness possible (i.e. 100% of the maximum) yields standardized effects that are limited to just above 0.2. To provide context, a widely used conventional set of standards for describing the substantive size of standardized effects sets the value of 0.2 as the benchmark for a "small" effect (see Jacob Cohen, *Statistical Power Analysis for the Behavioral Sciences*, Routledge, 2013). In contrast, the much larger standardized effects that increments of the other three performance metrics are able to generate get closer to the values of 0.5 and even 0.8 that serve as the conventional benchmarks for "medium" and "large" effects.

	Hum (v. Hum)	Algo (v. Algo)	Hum (v. Algo)	Algo (v. Hum)
Crime scenario				
Average rating	3.97 (0.02)	3.72 (0.02)	4.14 (0.02)	4.04 (0.02)
Loans scenario				
Average rating	4.24 (0.02)	4.08 (0.02)	4.36 (0.02)	4.27 (0.02)

Table S6. Average rating of DM in each condition, with standard errors clustered at the respondent level.

Proportion		
Crime scenario	*Loans scenario*	Statement
0.406 (0.007)	0.502 (0.008)	Algo DM has lowest Default/Crime rate
0.593 (0.007)	0.498 (0.008)	Human DM has lowest Default/Crime rate
0.528 (0.007)	0.579 (0.008)	Algo DM is Fairest
0.472 (0.007)	0.421 (0.008)	Human DM is Fairest
0.331 (0.007)	0.384 (0.007)	Algo DM is Dominant
0.398 (0.007)	0.303 (0.007)	Human DM is Dominant
0.270 (0.006)	0.313 (0.007)	Mixed Dominance

Table S7. Raw values of responses to direct survey question on DM preference. Standard errors shown in paranthesis. A dominant DM is one that is *both* fairer and results in a lower crime/default rate.

	Crime scenario		*Loans scenario*	
Believe Dominant DM to be...	Algo chosen	Human chosen	Algo chosen	Human chosen
Algo	0.564 (0.008)	0.436 (0.008)	0.540 (0.007)	0.460 (0.007)
Human	0.371 (0.008)	0.629 (0.008)	0.408 (0.009)	0.592 (0.009)
Mixed	0.466 (0.008)	0.534 (0.008)	0.475 (0.008)	0.525 (0.008)

Table S8. Proportion of the time that each DM is chosen, by the respondents' stated preferences. The first row corresponds to respondents who believe an Algorithmic DM to be dominant, etc. Standard errors given in parentheses. Correspondents to Figure 3. "Mixed" characterizes respondents who did not believe that either DM type was dominant.

Crime scenario				
Priority	Algo (v. Algo)	Hum (v. Hum)	Faceoff	All
Low crime rate	0.520 (0.013)	0.585 (0.012)	0.507 (0.013)	0.538 (0.007)
Low FPR	0.216 (0.010)	0.181 (0.010)	0.210 (0.010)	0.202 (0.006)
Fairness	0.264 (0.011)	0.233 (0.010)	0.283 (0.011)	0.260 (0.006)
Loans scenario				
Priority	Algo (v. Algo)	Hum (v. Hum)	Faceoff	All
Low default rate	0.534 (0.013)	0.647 (0.013)	0.568 (0.013)	0.584 (0.008)
Low FPR	0.197 (0.011)	0.147 (0.009)	0.187 (0.010)	0.177 (0.006)
Fairness	0.269 (0.012)	0.206 (0.011)	0.245 (0.011)	0.240 (0.007)

Table S9. Stated priorities of respondents versus the condition of the conjoint survey.

Crime scenario	All (1)	Algo (v. Algo) (2)	Hum (v. Hum) (3)	Faceoff (4)	Hum (v. Algo) (5)	Algo (v. Hum) (6)
Crime rate	0.0803	0.0703	0.1233	0.0552	0.0526	0.0592
	(0.0027)	(0.0046)	(0.0053)	(0.0038)	(0.0045)	(0.0047)
Unfairness	0.0013	0.0017	0.002	7e-04	6e-04	0.001
	(3e-04)	(5e-04)	(6e-04)	(3e-04)	(4e-04)	(6e-04)
MFPR	0.0398	0.0319	0.0528	0.037	0.0357	0.0392
	(0.0017)	(0.0027)	(0.0033)	(0.0029)	(0.0036)	(0.0038)
WFPR	0.0208	0.0205	0.0276	0.016	0.017	0.016
	(0.0012)	(0.002)	(0.0022)	(0.0017)	(0.0023)	(0.0023)
Loans scenario						
Default rate	0.0754	0.0657	0.1057	0.059	0.057	0.0615
	(0.0026)	(0.0042)	(0.0054)	(0.0043)	(0.0053)	(0.0054)
Unfairness	0.0016	0.0023	0.0016	0.0014	0.0017	0.0013
	(3e-04)	(7e-04)	(6e-04)	(5e-04)	(8e-04)	(7e-04)
MFPR	0.0527	0.0478	0.0571	0.054	0.0504	0.0589
	(0.002)	(0.0035)	(0.0036)	(0.0036)	(0.0047)	(0.0049)
WFPR	0.0283	0.025	0.0358	0.0254	0.0245	0.0279
	(0.0014)	(0.0023)	(0.0027)	(0.0023)	(0.003)	(0.0032)

Table S10. Average permutation variable importances for each data setting. Specifically, the values give the increase in log-loss obtained when the given feature is randomly permuted, averaged over 50 permutations each for 1,000 bootstrapped samples. We use a 3rd degree polynomial regression (with no interactions) using all four features. Within one data setting, all point estimates are significantly different at the $p = .05$ level. Note: The variable importance of crime/default rate, along with fairness, corresponds to hypotheses 6-8 in the pre-registered analysis. The importance of MFPR and WFPR is not pre-registered.

Additional Regression Results

Loans scenario

	Humans	Algos	Faceoff	Hum (v. Alg)	Alg (v. Hum)
Default rate	-0.050^{***}	-0.040^{***}	-0.038^{***}	-0.037^{***}	-0.038^{***}
	(0.001)	(0.001)	(0.001)	(0.002)	(0.002)
WFPR	-0.028^{***}	-0.024^{***}	-0.025^{***}	-0.024^{***}	-0.026^{***}
	(0.001)	(0.001)	(0.001)	(0.001)	(0.001)
MFPR	-0.036^{***}	-0.034^{***}	-0.036^{***}	-0.035^{***}	-0.037^{***}
	(0.001)	(0.001)	(0.001)	(0.002)	(0.001)
Constant	1.073^{***}	0.991^{***}	0.995^{***}	1.003^{***}	0.988^{***}
	(0.009)	(0.010)	(0.011)	(0.014)	(0.014)
Observations	28,760	27,720	28,320	14,160	14,160
R^2	0.168	0.121	0.122	0.116	0.130

Note: $^{*}p<0.1$; $^{**}p<0.05$; $^{***}p<0.01$

Table S11. Regression results split by condition and DM type for the Loans scenario. The dependent variable is the choice outcome. Corresponds to baseline experimental results in the pre-registered analysis.

Crime scenario

	Humans	Algos	Faceoff	Hum (v. Alg)	Alg (v. Hum)
Crime rate	-0.014^{***}	-0.011^{***}	-0.009^{***}	-0.009^{***}	-0.010^{***}
	(0.0003)	(0.0003)	(0.0003)	(0.0004)	(0.0004)
WFPR	-0.006^{***}	-0.006^{***}	-0.005^{***}	-0.005^{***}	-0.005^{***}
	(0.0002)	(0.0003)	(0.0003)	(0.0004)	(0.0004)
MFPR	-0.009^{***}	-0.007^{***}	-0.008^{***}	-0.008^{***}	-0.008^{***}
	(0.0003)	(0.0003)	(0.0003)	(0.0004)	(0.0004)
Constant	1.369^{***}	1.201^{***}	1.169^{***}	1.200^{***}	1.140^{***}
	(0.013)	(0.018)	(0.018)	(0.022)	(0.022)
Observations	32,660	31,680	31,340	15,670	15,670
R^2	0.173	0.110	0.099	0.095	0.104

Note: $^{*}p<0.1$; $^{**}p<0.05$; $^{***}p<0.01$

Table S12. Regression results split by condition and DM type for the Crime scenario. The dependent variable is the choice outcome. Corresponds to baseline experimental results in the pre-registered analysis.

Loans scenario

	Humans	Algos	Faceoff	Hum (v. Alg)	Alg (v. Hum)
	(1)	(2)	(3)	(4)	(5)
Fairness	−0.008***	−0.010***	−0.006***	−0.006***	−0.007***
	(0.001)	(0.001)	(0.001)	(0.002)	(0.002)
Constant	0.527***	0.535***	0.521***	0.542***	0.501***
	(0.004)	(0.004)	(0.004)	(0.008)	(0.008)
Observations	28,760	27,720	28,320	14,160	14,160
R^2	0.002	0.002	0.001	0.001	0.001

Note: *p<0.1; **p<0.05; ***p<0.01

Table S13. Regression results split by condition and DM type for the Loans scenario, and considering Fairness only. The dependent variable is the choice outcome. Corresponds to baseline experimental results in the pre-registered analysis.

Crime scenario

	Humans	Algos	Faceoff	Hum (v. Alg)	Alg (v. Hum)
	(1)	(2)	(3)	(4)	(5)
Fairness	−0.003***	−0.002***	−0.001***	−0.001*	−0.001***
	(0.0003)	(0.0003)	(0.0003)	(0.0004)	(0.0004)
Constant	0.536***	0.533***	0.516***	0.549***	0.482***
	(0.004)	(0.004)	(0.004)	(0.007)	(0.008)
Observations	32,660	31,680	31,340	15,670	15,670
R^2	0.003	0.002	0.001	0.0002	0.001

Note: *p<0.1; **p<0.05; ***p<0.01

Table S14. Regression results split by condition and DM type for the Crime scenario, and considering Fairness only. The dependent variable is the choice outcome. Corresponds to baseline experimental results in the pre-registered analysis.

	Crime scenario	*Loans scenario*
Unfairness	-0.002^{***}	-0.008^{***}
	(0.0002)	(0.001)
Crime/Default rate	-0.011^{***}	-0.042^{***}
	(0.0002)	(0.001)
Constant	0.867^{***}	0.740^{***}
	(0.006)	(0.004)
Observations	95,680	84,800
R^2	0.074	0.067

Note: *p<0.1; **p<0.05; ***p<0.01

Table S15. Regression results using all data, and only Crime/Default rate and Unfairness as predictors. The dependent variable is the choice outcome. Note: This regression was not pre-registered.

	Crime scenario	*Loans scenario*
Unfairness	-0.002^{***}	-0.008^{***}
	(0.0003)	(0.001)
MFPR + WFPR	-0.007^{***}	-0.030^{***}
	(0.0001)	(0.0005)
Constant	0.936^{***}	0.832^{***}
	(0.008)	(0.006)
Observations	71,700	63,636
R^2	0.066	.086

Note: *p<0.1; **p<0.05; ***p<0.01

Table S16. Regression results for data filtered to Unfairness ≤ 20 for Crime scenario, and Unfairness ≤ 5 for Loans scenario. Figure S6 validates the linear effect assumption. The dependent variable is the choice outcome. Note: This regression was not pre-registered.

	Crime scenario			*Loans scenario*		
	(1)	(2)	(3)	(4)	(5)	(6)
C/D rate	-0.011^{***}	-0.011^{***}		-0.040^{***}	-0.040^{***}	
	(0.0003)	(0.0003)		(0.001)	(0.001)	
WFPR	-0.006^{***}	-0.006^{***}		-0.024^{***}	-0.024^{***}	
	(0.0003)	(0.0003)		(0.001)	(0.001)	
MFPR	-0.007^{***}	-0.007^{***}		-0.034^{***}	-0.034^{***}	
	(0.0003)	(0.0003)		(0.001)	(0.001)	
Unfairness		-0.002^{***}	-0.002^{***}		-0.010^{***}	-0.010^{***}
		(0.0003)	(0.0003)		(0.001)	(0.001)
Human	0.168^{***}	0.169^{***}	0.003	0.082^{***}	0.078^{***}	-0.007
	(0.022)	(0.023)	(0.006)	(0.014)	(0.015)	(0.006)
C/D rate:Human	-0.003^{***}	-0.003^{***}		-0.010^{***}	-0.010^{***}	
	(0.0004)	(0.0004)		(0.002)	(0.002)	
WFPR:Human	-0.001^{*}	-0.001^{*}		-0.004^{***}	-0.004^{***}	
	(0.0004)	(0.0004)		(0.002)	(0.002)	
MFPR:Human	-0.002^{***}	-0.002^{***}		-0.002	-0.002	
	(0.0004)	(0.0004)		(0.002)	(0.002)	
Unfairness:Human		-0.0001	-0.0002		0.001	0.002
		(0.0004)	(0.0004)		(0.002)	(0.002)
Constant	1.201^{***}	1.233^{***}	0.533^{***}	0.991^{***}	1.024^{***}	0.535^{***}
	(0.018)	(0.018)	(0.004)	(0.010)	(0.011)	(0.004)
Observations	64,340	64,340	64,340	56,480	56,480	56,480
R^2	0.142	0.144	0.002	0.145	0.147	0.002

Note: *p$<$0.1; **p$<$0.05; ***p$<$0.01. C/D rate = Crime/Default rate.

Table S17. Testing contextual performance preferences using all data from non-Faceoff conditions. Corresponds to Hypotheses 1 and 4 in the pre-registered analysis. The dependent variable is the choice outcome.

	Crime scenario			Loans scenario		
	(1)	(2)	(3)	(4)	(5)	(6)
C/D rate		−0.009***	−0.009***		−0.038***	−0.038***
		(0.0003)	(0.0003)		(0.001)	(0.001)
WFPR		−0.005***	−0.005***		−0.025***	−0.025***
		(0.0003)	(0.0003)		(0.001)	(0.001)
MFPR		−0.008***	−0.008***		−0.036***	−0.036***
		(0.0003)	(0.0003)		(0.001)	(0.001)
Human	0.076***	0.078***	0.078***	0.043***	0.042***	0.042***
	(0.010)	(0.010)	(0.010)	(0.010)	(0.009)	(0.009)
Unfairness			−0.001***			−0.007***
			(0.0003)			(0.001)
Constant	0.462***	1.131***	1.146***	0.478***	0.974***	0.999***
	(0.005)	(0.020)	(0.020)	(0.005)	(0.012)	(0.013)
Observations	31,340	31,340	31,340	28,320	28,320	28,320
R^2	0.006	0.105	0.105	0.002	0.124	0.125

Note: *p<0.1; **p<0.05; ***p<0.01. C/D rate = Crime/Default rate.

Table S18. Testing human v. algorithm procedural bias using data from the Faceoff condition. Corresponds to Hypothesis 2 in the pre-registered analysis. The dependent variable is the choice outcome.

Crime scenario

	Faceoff	Faceoff	Human	Human	Algo	Algo
	(1)	(2)	(3)	(4)	(5)	(6)
Faceoff			−0.169***	−0.192***	−0.061**	−0.073**
			(0.025)	(0.026)	(0.028)	(0.029)
Crime rate	−0.010***	−0.010***	−0.014***	−0.014***	−0.011***	−0.011***
	(0.0004)	(0.0004)	(0.0003)	(0.0003)	(0.0003)	(0.0003)
WFPR	−0.005***	−0.005***	−0.006***	−0.006***	−0.006***	−0.006***
	(0.0004)	(0.0004)	(0.0002)	(0.0002)	(0.0003)	(0.0003)
MFPR	−0.008***	−0.008***	−0.009***	−0.009***	−0.007***	−0.007***
	(0.0004)	(0.0004)	(0.0003)	(0.0003)	(0.0003)	(0.0003)
Unfairness		−0.001***		−0.002***		−0.002***
		(0.0004)		(0.0003)		(0.0003)
Human	0.060**	0.051**				
	(0.024)	(0.025)				
Crime rate:Human	0.001	0.001				
	(0.0004)	(0.0004)				
WFPR:Human	−0.0002	−0.0002				
	(0.0004)	(0.0004)				
MFPR:Human	0.0003	0.0003				
	(0.0004)	(0.0004)				
Unfairness:Human		0.001				
		(0.001)				
Faceoff:Crime rate			0.005***	0.005***	0.001*	0.001*
			(0.0005)	(0.0005)	(0.0005)	(0.0005)
Faceoff:WFPR			0.001***	0.001***	0.001	0.001
			(0.0004)	(0.0004)	(0.0004)	(0.0004)
Faceoff:MFPR			0.001***	0.001***	−0.001*	−0.001
			(0.0005)	(0.0005)	(0.0005)	(0.0005)
Faceoff:Unfairness				0.002***		0.001*
				(0.0005)		(0.0005)
Constant	1.140***	1.160***	1.369***	1.403***	1.201***	1.233***
	(0.022)	(0.023)	(0.013)	(0.014)	(0.018)	(0.018)
Observations	31,340	31,340	48,330	48,330	47,350	47,350
R^2	0.105	0.105	0.149	0.151	0.109	0.111

Note: *p<0.1; **p<0.05; ***p<0.01

Table S19. Testing human v. algorithm performance-based preferences in the Crime scenario. Corresponds to Hypothesis 3 in the pre-registered analysis. In columns 3-4, the data is comprised of all rows (across Faceoff and non-Faceoff conditions) where the DM is human. Similarly, in columns 5-6, the data is comprised of all rows (across Faceoff and non-Faceoff conditions) where the DM is an algorithm. The dependent variable is the choice outcome.

Loans scenario

	Faceoff	Faceoff	Human	Human	Algo	Algo
	(1)	(2)	(3)	(4)	(5)	(6)
Faceoff			−0.070***	−0.079***	−0.003	−0.006
			(0.017)	(0.018)	(0.018)	(0.019)
Default rate	−0.038***	−0.038***	−0.050***	−0.050***	−0.040***	−0.040***
	(0.002)	(0.002)	(0.001)	(0.001)	(0.001)	(0.001)
WFPR	−0.026***	−0.026***	−0.028***	−0.028***	−0.024***	−0.024***
	(0.001)	(0.001)	(0.001)	(0.001)	(0.001)	(0.001)
MFPR	−0.037***	−0.038***	−0.036***	−0.036***	−0.034***	−0.034***
	(0.001)	(0.001)	(0.001)	(0.001)	(0.001)	(0.001)
Unfairness		−0.008***		−0.008***		−0.010***
		(0.002)		(0.001)		(0.001)
Human	0.015	0.005				
	(0.018)	(0.019)				
Default rate:Human	0.001	0.001				
	(0.002)	(0.002)				
WFPR:Human	0.002	0.002				
	(0.002)	(0.002)				
MFPR:Human	0.003	0.003				
	(0.002)	(0.002)				
Unfairness:Human		0.003				
		(0.002)				
Faceoff:Default rate			0.012***	0.013***	0.002	0.001
			(0.002)	(0.002)	(0.002)	(0.002)
Faceoff:WFPR			0.004**	0.005**	−0.001	−0.001
			(0.002)	(0.002)	(0.002)	(0.002)
Faceoff:MFPR			0.001	0.001	−0.003*	−0.004*
			(0.002)	(0.002)	(0.002)	(0.002)
Faceoff:Unfairness				0.002		0.001
				(0.002)		(0.002)
Constant	0.988***	1.018***	1.073***	1.102***	0.991***	1.024***
	(0.014)	(0.016)	(0.009)	(0.009)	(0.010)	(0.011)
Observations	28,320	28,320	42,920	42,920	41,880	41,880
R^2	0.124	0.126	0.151	0.152	0.124	0.126

Note: *p<0.1; **p<0.05; ***p<0.01

Table S20. Testing human v. algorithm performance-based preferences in the Loans scenario. Corresponds to Hypothesis 3 in the pre-registered analysis. In columns 3-4, the data is comprised of all rows (across Faceoff and non-Faceoff conditions) where the DM is human. Similarly, in columns 5-6, the data is comprised of all rows (across Faceoff and non-Faceoff conditions) where the DM is an algorithm. The dependent variable is the choice outcome.

Crime scenario

	Faceoff	Human	Human	Algo	Algo
	(1)	(2)	(3)	(4)	(5)
Faceoff		0.013	−0.192***	−0.051***	−0.073**
		(0.009)	(0.026)	(0.009)	(0.029)
Crime rate			−0.014***		−0.011***
			(0.0003)		(0.0003)
WFPR			−0.006***		−0.006***
			(0.0002)		(0.0003)
MFPR			−0.009***		−0.007***
			(0.0003)		(0.0003)
Unfairness	−0.001***	−0.003***	−0.002***	−0.002***	−0.002***
	(0.0004)	(0.0003)	(0.0003)	(0.0003)	(0.0003)
Human	0.067***				
	(0.013)				
Unfairness:Human	0.001				
	(0.001)				
Faceoff:Crime rate			0.005***		0.001*
			(0.0005)		(0.0005)
Faceoff:WFPR			0.001***		0.001
			(0.0004)		(0.0004)
Faceoff:MFPR			0.001***		−0.001
			(0.0005)		(0.0005)
Faceoff:Unfairness		0.002***	0.002***	0.001*	0.001*
		(0.001)	(0.0005)	(0.001)	(0.0005)
Constant	0.482***	0.536***	1.403***	0.533***	1.233***
	(0.008)	(0.004)	(0.014)	(0.004)	(0.018)
Observations	31,340	48,330	48,330	47,350	47,350
R^2	0.006	0.003	0.151	0.003	0.111

Note: *p<0.1; **p<0.05; ***p<0.01

Table S21. Testing differences in fairness expectations for humans, depending on the matchup, in the Crime scenario. Corresponds to Hypothesis 5 in the pre-registsered analysis. In columns 2-3, the data is comprised of all rows (across Faceoff and non-Faceoff conditions) where the DM is human. Similarly, in columns 2-3, the data is comprised of all rows (across Faceoff and non-Faceoff conditions) where the DM is an algorithm. The dependent variable is the choice outcome.

Loans scenario

	Faceoff	Human	Human	Algo	Algo
	(1)	(2)	(3)	(4)	(5)
Faceoff		0.013	-0.192^{***}	-0.051^{***}	-0.073^{**}
		(0.009)	(0.026)	(0.009)	(0.029)
Default rate			-0.014^{***}		-0.011^{***}
			(0.0003)		(0.0003)
WFPR			-0.006^{***}		-0.006^{***}
			(0.0002)		(0.0003)
MFPR			-0.009^{***}		-0.007^{***}
			(0.0003)		(0.0003)
Unfairness	-0.007^{***}	-0.003^{***}	-0.002^{***}	-0.002^{***}	-0.002^{***}
	(0.002)	(0.0003)	(0.0003)	(0.0003)	(0.0003)
Human	0.040^{***}				
	(0.013)				
Unfairness:Human	0.001				
	(0.002)				
Faceoff:Default rate			0.005^{***}		0.001^{*}
			(0.0005)		(0.0005)
Faceoff:WFPR			0.001^{***}		0.001
			(0.0004)		(0.0004)
Faceoff:MFPR			0.001^{***}		-0.001
			(0.0005)		(0.0005)
Faceoff:Unfairness		0.002^{***}	0.002^{***}	0.001^{*}	0.001^{*}
		(0.001)	(0.0005)	(0.001)	(0.0005)
Constant	0.501^{***}	0.536^{***}	1.403^{***}	0.533^{***}	1.233^{***}
	(0.008)	(0.004)	(0.014)	(0.004)	(0.018)
Observations	28,320	48,330	48,330	47,350	47,350
R^2	0.003	0.003	0.151	0.003	0.111

Note: *p<0.1; **p<0.05; ***p<0.01

Table S22. Testing differences in fairness expectations for humans, depending on the matchup, in the Loans scenario. Corresponds to Hypothesis 5 in the pre-registsered analysis. In columns 2-3, the data is comprised of all rows (across Faceoff and non-Faceoff conditions) where the DM is human. Similarly, in columns 2-3, the data is comprised of all rows (across Faceoff and non-Faceoff conditions) where the DM is an algorithm. The dependent variable is the choice outcome.

Crime scenario

	Hum (v. Hum)	Algo (v. Algo)	Hum (v. Algo)	Algo (v. Hum)
Crime/Default rate	-0.031^{***}	-0.024^{***}	-0.022^{***}	-0.021^{***}
	(0.001)	(0.001)	(0.001)	(0.001)
WFPR	-0.016^{***}	-0.015^{***}	-0.014^{***}	-0.014^{***}
	(0.001)	(0.001)	(0.001)	(0.001)
MFPR	-0.025^{***}	-0.020^{***}	-0.019^{***}	-0.019^{***}
	(0.001)	(0.001)	(0.001)	(0.001)
Unfairness	-0.010^{***}	-0.010^{***}	-0.005^{***}	-0.007^{***}
	(0.001)	(0.001)	(0.001)	(0.001)
Constant	6.263^{***}	5.591^{***}	5.847^{***}	5.738^{***}
	(0.049)	(0.057)	(0.066)	(0.067)
Observations	32,660	31,680	15,670	15,670
R^2	0.123	0.073	0.068	0.068

Note: *p<0.1; **p<0.05; ***p<0.01

Table S23. Basic regression results predicting DM rating in the Crime scenario. Note: this analysis is unregistered.

Loans scenario

	Hum (v. Hum)	Algo (v. Algo)	Hum (v. Algo)	Algo (v. Hum)
Crime/Default rate	-0.132^{***}	-0.100^{***}	-0.097^{***}	-0.091^{***}
	(0.004)	(0.004)	(0.005)	(0.005)
WFPR	-0.067^{***}	-0.064^{***}	-0.059^{***}	-0.072^{***}
	(0.003)	(0.004)	(0.004)	(0.004)
MFPR	-0.103^{***}	-0.097^{***}	-0.102^{***}	-0.104^{***}
	(0.003)	(0.004)	(0.005)	(0.005)
Unfairness	-0.039^{***}	-0.040^{***}	-0.030^{***}	-0.033^{***}
	(0.004)	(0.004)	(0.005)	(0.005)
Constant	5.882^{***}	5.517^{***}	5.758^{***}	5.731^{***}
	(0.036)	(0.042)	(0.047)	(0.048)
Observations	28,760	27,720	14,160	14,160
R^2	0.140	0.090	0.097	0.100

Note: *p<0.1; **p<0.05; ***p<0.01

Table S24. Basic regression results predicting DM rating in the Loans scenario. Note: this analysis is unregistered.

Regression Results for Heterogeneity

Crime scenario

	All data		Faceoff data	
	Non-white	White	Non-white	White
	(1)	(2)	(3)	(4)
Crime rate	−0.010***	−0.012***	−0.009***	−0.010***
	(0.0003)	(0.0002)	(0.001)	(0.0004)
WFPR	−0.005***	−0.006***	−0.004***	−0.006***
	(0.0002)	(0.0002)	(0.0004)	(0.0003)
MFPR	−0.009***	−0.007***	−0.008***	−0.007***
	(0.0003)	(0.0002)	(0.001)	(0.0004)
Fairness	−0.002***	−0.002***	−0.001***	−0.001***
	(0.0003)	(0.0002)	(0.0005)	(0.0004)
Human			0.069***	0.083***
			(0.016)	(0.012)
Constant	1.235***	1.297***	1.121***	1.161***
	(0.017)	(0.012)	(0.033)	(0.026)
Observations	34,820	60,860	11,800	19,540
R^2	0.115	0.136	0.098	0.111

Note: *p<0.1; **p<0.05; ***p<0.01

Table S25. Results for White and Non-white respondents in the Crime scenario. The left two columns show aggregate results across all conditions. The right two columns show the Faceoff condition only. In all columns, the coefficients on WFPR and MFPR are significantly different ($p < .005$). The dependent variable is the choice outcome.

Loans scenario

	All data		Faceoff data	
	Non-white	White	Non-white	White
	(1)	(2)	(3)	(4)
Default rate	−0.035***	−0.047***	−0.031***	−0.042***
	(0.001)	(0.001)	(0.002)	(0.002)
WFPR	−0.021***	−0.029***	−0.019***	−0.028***
	(0.001)	(0.001)	(0.002)	(0.001)
MFPR	−0.040***	−0.033***	−0.040***	−0.034***
	(0.001)	(0.001)	(0.002)	(0.001)
Fairness	−0.009***	−0.008***	−0.007***	−0.007***
	(0.001)	(0.001)	(0.002)	(0.001)
Human			0.039***	0.043***
			(0.015)	(0.011)
Constant	1.012***	1.071***	0.958***	1.023***
	(0.011)	(0.008)	(0.024)	(0.016)
Observations	31,620	53,180	10,180	18,140
R^2	0.121	0.151	0.107	0.139

Note: *p<0.1; **p<0.05; ***p<0.01

Table S26. Results for White and Non-white respondents in the Loans scenario. The left two columns show aggregate results across all conditions. The right two columns show the Faceoff condition only. In all columns, the coefficients on WFPR and MFPR are significantly different ($p < .005$). The dependent variable is the choice outcome.

Crime scenario

	All data			Faceoff data		
	Democrat	Indep.	Republican	Democrat	Indep.	Republican
	(1)	(2)	(3)	(4)	(5)	(6)
Crime rate	−0.010***	−0.012***	−0.012***	−0.009***	−0.010***	−0.009***
	(0.0003)	(0.0003)	(0.0004)	(0.0005)	(0.001)	(0.001)
WFPR	−0.005***	−0.006***	−0.006***	−0.005***	−0.005***	−0.005***
	(0.0002)	(0.0002)	(0.0003)	(0.0004)	(0.0005)	(0.001)
MFPR	−0.009***	−0.008***	−0.006***	−0.009***	−0.008***	−0.006***
	(0.0003)	(0.0003)	(0.0003)	(0.0005)	(0.0005)	(0.001)
Fairness	−0.002***	−0.002***	−0.002***	−0.001***	−0.001**	−0.0005
	(0.0003)	(0.0003)	(0.0003)	(0.0004)	(0.0005)	(0.001)
Human				0.045***	0.079***	0.127***
				(0.015)	(0.016)	(0.020)
Constant	1.251***	1.320***	1.247***	1.172***	1.186***	1.051***
	(0.016)	(0.015)	(0.021)	(0.031)	(0.033)	(0.044)
Observations	37,840	33,800	24,040	12,780	10,660	7,900
R^2	0.116	0.146	0.123	0.102	0.122	0.096

Note: *p<0.1; **p<0.05; ***p<0.01. Indep = Independent/Other

Table S27. Results across stated political party for respondents in the Crime scenario. The three leftmost columns show aggregate results across all conditions. The three rightmost columns show the Faceoff condition only. The dependent variable is the choice outcome.

Loans scenario

	All data			Faceoff data		
	Democrat	Indep.	Republican	Democrat	Indep.	Republican
	(1)	(2)	(3)	(4)	(5)	(6)
Default rate	−0.038***	−0.044***	−0.047***	−0.036***	−0.037***	−0.042***
	(0.001)	(0.001)	(0.001)	(0.002)	(0.002)	(0.002)
WFPR	−0.024***	−0.026***	−0.029***	−0.022***	−0.025***	−0.029***
	(0.001)	(0.001)	(0.001)	(0.002)	(0.002)	(0.002)
MFPR	−0.039***	−0.036***	−0.030***	−0.039***	−0.036***	−0.031***
	(0.001)	(0.001)	(0.001)	(0.002)	(0.002)	(0.002)
Fairness	−0.009***	−0.010***	−0.006***	−0.006***	−0.009***	−0.007***
	(0.001)	(0.001)	(0.001)	(0.002)	(0.002)	(0.002)
Human				0.033**	0.036**	0.064***
				(0.014)	(0.015)	(0.019)
Constant	1.037***	1.066***	1.046***	0.986***	1.009***	1.006***
	(0.010)	(0.011)	(0.013)	(0.020)	(0.023)	(0.028)
Observations	33,580	29,900	21,320	11,260	10,180	6,880
R^2	0.132	0.145	0.143	0.122	0.124	0.137

Note: *p<0.1; **p<0.05; ***p<0.01. Indep = Independent/Other

Table S28. Results across stated political party for respondents in the Loans scenario. The three leftmost columns show aggregate results across all conditions. The three rightmost columns show the Faceoff condition only. The dependent variable is the choice outcome.

Crime scenario

	All data		Faceoff data	
	No college degree	College degree	No college degree	College degree
	(1)	(2)	(3)	(4)
Crime rate	−0.011***	−0.012***	−0.009***	−0.010***
	(0.0002)	(0.0003)	(0.0004)	(0.001)
WFPR	−0.006***	−0.006***	−0.005***	−0.005***
	(0.0002)	(0.0002)	(0.0004)	(0.0004)
MFPR	−0.008***	−0.008***	−0.008***	−0.008***
	(0.0002)	(0.0003)	(0.0004)	(0.0005)
Fairness	−0.002***	−0.002***	−0.001***	−0.001*
	(0.0002)	(0.0003)	(0.0004)	(0.0004)
Human			0.088***	0.060***
			(0.012)	(0.016)
Constant	1.258***	1.307***	1.120***	1.192***
	(0.012)	(0.016)	(0.026)	(0.032)
Observations	61,460	34,220	19,640	11,700
R^2	0.121	0.139	0.100	0.115

Note: *p<0.1; **p<0.05; ***p<0.01

Table S29. Results across education level for respondents in the Crime scenario. The two leftmost columns show aggregate results across all conditions. The two rightmost columns show the Faceoff condition only. The dependent variable is the choice outcome.

Loans scenario

	All data		Faceoff data	
	No college degree	College degree	No college degree	College degree
	(1)	(2)	(3)	(4)
Default rate	−0.039***	−0.049***	−0.034***	−0.046***
	(0.001)	(0.001)	(0.002)	(0.002)
WFPR	−0.027***	−0.024***	−0.024***	−0.027***
	(0.001)	(0.001)	(0.001)	(0.002)
MFPR	−0.034***	−0.038***	−0.034***	−0.040***
	(0.001)	(0.001)	(0.001)	(0.002)
Fairness	−0.009***	−0.008***	−0.007***	−0.007***
	(0.001)	(0.001)	(0.001)	(0.002)
Human			0.048***	0.031**
			(0.011)	(0.015)
Constant	1.031***	1.085***	0.961***	1.073***
	(0.008)	(0.010)	(0.017)	(0.020)
Observations	55,220	29,580	18,480	9,840
R^2	0.127	0.161	0.110	0.159

Note: *p<0.1; **p<0.05; ***p<0.01

Table S30. Results across education level for respondents in the Loans scenario. The two leftmost columns show aggregate results across all conditions. The two rightmost columns show the Faceoff condition only. The dependent variable is the choice outcome.

Crime scenario

	All data		Faceoff data	
	AI Optimist	AI Pessimist	AI Optimist	AI Pessimist
	(1)	(2)	(3)	(4)
Crime rate	−0.011***	−0.011***	−0.010***	−0.009***
	(0.0001)	(0.0002)	(0.0003)	(0.0004)
WFPR	−0.006***	−0.006***	−0.005***	−0.005***
	(0.0001)	(0.0003)	(0.0003)	(0.0004)
MFPR	−0.008***	−0.008***	−0.008***	−0.008***
	(0.0001)	(0.0003)	(0.0003)	(0.0004)
Fairness	−0.002***	−0.002***	−0.001***	−0.001**
	(0.0002)	(0.0003)	(0.0003)	(0.001)
Human			0.057***	0.134***
			(0.006)	(0.010)
Constant	1.271***	1.287***	1.149***	1.138***
	(0.008)	(0.014)	(0.015)	(0.025)
Observations	70,860	24,820	23,000	8,340
R^2	0.126	0.130	0.101	0.122

Note: *p<0.1; **p<0.05; ***p<0.01

Table S31. Results across respondents' views on AI for respondents in the Crime scenario. The two leftmost columns show aggregate results across all conditions. The two rightmost columns show the Faceoff condition only. The dependent variable is the choice outcome. To define "AI pessimists" and "AI optimists," we created an AI affinity index by combining respondents' answers to two direct question prompts: "Thinking about the possibility that computers and robots could do most of the work currently done by humans... (1) How ENTHUSIASTIC are you, if at all, about this possibility for society as a whole? (2) How WORRIED are you, if at all, about this possibility for society as a whole?" Answer options included: Very enthusiastic/worried, Somewhat enthusiastic/worried, Not too enthusiastic/worried, and Not at all enthusiastic/worried. These answer options were mapped onto a numeric scale from 1-4 (with the scale reversed for the first question), and respondents' values were then added to create the affinity index. Respondents who scored below the median value on this AI affinity index were designated "AI pessimists," and all others designated "AI optimists."

Loans scenario

	All data		Faceoff data	
	AI Optimist	AI Pessimist	AI Optimist	AI Pessimist
	(1)	(2)	(3)	(4)
Default rate	−0.042***	−0.043***	−0.037***	−0.040***
	(0.001)	(0.001)	(0.001)	(0.002)
WFPR	−0.025***	−0.030***	−0.023***	−0.031***
	(0.001)	(0.001)	(0.001)	(0.002)
MFPR	−0.035***	−0.038***	−0.035***	−0.039***
	(0.001)	(0.001)	(0.001)	(0.002)
Fairness	−0.008***	−0.010***	−0.006***	−0.010***
	(0.001)	(0.001)	(0.001)	(0.002)
Human			0.032***	0.075***
			(0.006)	(0.011)
Constant	1.036***	1.089***	0.982***	1.056***
	(0.006)	(0.011)	(0.011)	(0.020)
Observations	63,700	21,100	21,500	6,820
R^2	0.133	0.154	0.117	0.156

Note: *p<0.1; **p<0.05; ***p<0.01.

Table S32. Results across respondents' views on AI for respondents in the Loans scenario. The two leftmost columns show aggregate results across all conditions. The two rightmost columns show the Faceoff condition only. The dependent variable is the choice outcome. To define "AI pessimists" and "AI optimists," we created an AI affinity index by combining respondents' answers to two direct question prompts: "Thinking about the possibility that computers and robots could do most of the work currently done by humans... (1) How ENTHUSIASTIC are you, if at all, about this possibility for society as a whole? (2) How WORRIED are you, if at all, about this possibility for society as a whole?" Answer options included: Very enthusiastic/worried, Somewhat enthusiastic/worried, Not too enthusiastic/worried, and Not at all enthusiastic/worried. These answer options were mapped onto a numeric scale from 1-4 (with the scale reversed for the first question), and respondents' values were then added to create the affinity index. Respondents who scored below the median value on this AI affinity index were designated "AI pessimists," and all others designated "AI optimists."

Crime scenario

	All data			Faceoff data		
	Fairness	Low crime rate	Low FPR	Fairness	Low crime rate	Low FPR
	(1)	(2)	(3)	(4)	(5)	(6)
Crime rate	−0.008***	−0.014***	−0.008***	−0.006***	−0.012***	−0.007***
	(0.0003)	(0.0002)	(0.0004)	(0.001)	(0.0004)	(0.001)
WFPR	−0.005***	−0.005***	−0.007***	−0.005***	−0.005***	−0.006***
	(0.0003)	(0.0002)	(0.0003)	(0.001)	(0.0004)	(0.001)
MFPR	−0.009***	−0.007***	−0.009***	−0.009***	−0.007***	−0.008***
	(0.0004)	(0.0002)	(0.0004)	(0.001)	(0.0004)	(0.001)
Fairness	−0.002***	−0.002***	−0.003***	−0.002***	−0.0002	−0.002***
	(0.0003)	(0.0002)	(0.0004)	(0.001)	(0.0004)	(0.001)
Human				0.090***	0.076***	0.064***
				(0.019)	(0.013)	(0.021)
Constant	1.197***	1.322***	1.250***	1.061***	1.194***	1.142***
	(0.021)	(0.012)	(0.024)	(0.041)	(0.027)	(0.046)
Observations	24,840	51,500	19,340	8,860	15,900	6,580
R^2	0.097	0.160	0.110	0.084	0.132	0.095

Note: *p<0.1; **p<0.05; ***p<0.01

Table S33. Results across respondents' stated priority in the Crime scenario. The two leftmost columns show aggregate results across all conditions. The two rightmost columns show the Faceoff condition only. The dependent variable is the choice outcome.

Loans scenario

	All data			Faceoff data		
	Fairness	Low default rate	Low FPR	Fairness	Low default rate	Low FPR
	(1)	(2)	(3)	(4)	(5)	(6)
Default rate	−0.023***	−0.055***	−0.028***	−0.022***	−0.050***	−0.024***
	(0.001)	(0.001)	(0.002)	(0.002)	(0.002)	(0.003)
WFPR	−0.025***	−0.025***	−0.028***	−0.023***	−0.025***	−0.026***
	(0.001)	(0.001)	(0.001)	(0.002)	(0.001)	(0.003)
MFPR	−0.039***	−0.032***	−0.042***	−0.038***	−0.034***	−0.040***
	(0.002)	(0.001)	(0.002)	(0.003)	(0.001)	(0.003)
Fairness	−0.010***	−0.007***	−0.009***	−0.007***	−0.006***	−0.011***
	(0.001)	(0.001)	(0.002)	(0.002)	(0.001)	(0.003)
Human				0.043**	0.049***	0.016
				(0.019)	(0.011)	(0.022)
Constant	0.974***	1.087***	1.023***	0.922***	1.037***	0.979***
	(0.015)	(0.008)	(0.015)	(0.030)	(0.016)	(0.031)
Observations	20,320	49,500	14,980	6,940	16,080	5,300
R^2	0.101	0.172	0.123	0.092	0.159	0.104

Note: *p<0.1; **p<0.05; ***p<0.01

Table S34. Results across respondents' stated priority in the Loans scenario. The two leftmost columns show aggregate results across all conditions. The two rightmost columns show the Faceoff condition only. The dependent variable is the choice outcome.

Crime scenario

	Non-Faceoff	Non-Faceoff	Faceoff	Faceoff	Faceoff	Faceoff
Crime rate	−0.011***			−0.010***	−0.010***	
	(0.0004)			(0.0004)	(0.0005)	
WFPR	−0.006***			−0.006***	−0.006***	
	(0.0003)			(0.0003)	(0.0004)	
MFPR	−0.007***			−0.007***	−0.008***	
	(0.0004)			(0.0004)	(0.0004)	
Unfairness		−0.002***				−0.001***
		(0.0004)				(0.001)
Human	0.138***	0.003	0.079***	0.083***	0.059**	0.065***
	(0.027)	(0.007)	(0.013)	(0.012)	(0.030)	(0.016)
Nonwhite	−0.117***	−0.002	0.004	−0.045	−0.052	0.004
	(0.037)	(0.008)	(0.011)	(0.041)	(0.046)	(0.016)
Crime rate:Human	−0.003***				0.001	
	(0.001)				(0.001)	
WFPR:Human	−0.001*				−0.0002	
	(0.0004)				(0.001)	
MFPR:Human	−0.001				0.0005	
	(0.0005)				(0.001)	
Crime rate:Nonwhite	0.002***			0.001**	0.001*	
	(0.001)			(0.001)	(0.001)	
WFPR:Nonwhite	0.002***			0.002***	0.002**	
	(0.001)			(0.001)	(0.001)	
MFPR:Nonwhite	−0.0005			−0.001*	−0.001	
	(0.001)			(0.001)	(0.001)	
Unfairness:Human		−0.0002				0.001
		(0.001)				(0.001)
Unfairness:Nonwhite		0.0001				−0.00004
		(0.001)				(0.001)
Human:Nonwhite	0.086*	−0.0002	−0.007	−0.014	0.0003	0.004
	(0.046)	(0.012)	(0.021)	(0.020)	(0.049)	(0.027)
Crime rate:Human:Nonwhite	0.0001				−0.00002	
	(0.001)				(0.001)	
WFPR:Human:Nonwhite	0.0003				−0.00004	
	(0.001)				(0.001)	
MFPR:Human:Nonwhite	−0.003***				−0.0004	
	(0.001)				(0.001)	
Unfairness:Human:Nonwhite		−0.00002				−0.001
		(0.001)				(0.001)
Constant	1.242***	0.534***	0.460***	1.148***	1.160***	0.480***
	(0.021)	(0.005)	(0.006)	(0.025)	(0.028)	(0.010)
Observations	64,340	64,340	31,340	31,340	31,340	31,340
R^2	0.144	0.002	0.006	0.106	0.106	0.006

Note: *p<0.1; **p<0.05; ***p<0.01.

Table S35. Heterogeneous results by race (white v. non-white) in the Crime scenario. Corresponds to Hypotheses 1-5 in the pre-registered analysis. The dependent variable is the choice outcome.

Loans scenario

	Non-Faceoff	Non-Faceoff	Faceoff	Faceoff	Faceoff	Faceoff
Default rate	−0.044***			−0.042***	−0.043***	
	(0.002)			(0.002)	(0.002)	
WFPR	−0.028***			−0.028***	−0.031***	
	(0.001)			(0.001)	(0.002)	
MFPR	−0.031***			−0.034***	−0.035***	
	(0.001)			(0.001)	(0.002)	
Unfairness		−0.010***				−0.008***
		(0.002)				(0.002)
Human	0.072***	−0.016**	0.046***	0.043***	0.001	0.039**
	(0.017)	(0.008)	(0.012)	(0.011)	(0.022)	(0.016)
Nonwhite	−0.074***	−0.005	0.004	−0.069***	−0.092***	−0.006
	(0.022)	(0.009)	(0.010)	(0.027)	(0.031)	(0.016)
Default rate:Human	−0.010***				0.002	
	(0.002)				(0.002)	
WFPR:Human	−0.002				0.005**	
	(0.002)				(0.002)	
MFPR:Human	−0.002				0.002	
	(0.002)				(0.002)	
Default rate:Nonwhite	0.011***			0.011***	0.012***	
	(0.003)			(0.003)	(0.003)	
WFPR:Nonwhite	0.010***			0.010***	0.014***	
	(0.002)			(0.002)	(0.003)	
MFPR:Nonwhite	−0.007***			−0.006**	−0.007**	
	(0.003)			(0.002)	(0.003)	
Unfairness:Human		0.005**				0.002
		(0.002)				(0.003)
Unfairness:Nonwhite		0.001				0.003
		(0.003)				(0.004)
Human:Nonwhite	0.026	0.022*	−0.008	−0.003	0.043	0.003
	(0.029)	(0.013)	(0.020)	(0.019)	(0.038)	(0.027)
Default rate:Human:Nonwhite	0.001				−0.002	
	(0.004)				(0.004)	
WFPR:Human:Nonwhite	−0.006**				−0.009**	
	(0.003)				(0.004)	
MFPR:Human:Nonwhite	0.0004				0.002	
	(0.003)				(0.004)	
Unfairness:Human:Nonwhite		−0.006*				−0.004
		(0.004)				(0.005)
Constant	1.019***	0.536***	0.477***	0.999***	1.020***	0.503***
	(0.012)	(0.006)	(0.006)	(0.015)	(0.017)	(0.009)
Observations	56,480	56,480	28,320	28,320	28,320	28,320
R^2	0.147	0.002	0.002	0.126	0.127	0.003

Note: *p<0.1; **p<0.05; ***p<0.01.

Table S36. Heterogeneous results by race (white v. non-white) in the Loans scenario. Corresponds to Hypotheses 1-5 in the pre-registered analysis. The dependent variable is the choice outcome.

Crime scenario

	Non-Faceoff	Faceoff
	(1)	(2)
Crime rate	−0.009***	−0.009***
	(0.001)	(0.001)
WFPR	−0.005***	−0.005***
	(0.0004)	(0.001)
MFPR	−0.007***	−0.008***
	(0.0005)	(0.001)
Human	0.207***	0.070*
	(0.037)	(0.038)
Indep/Other	0.127***	0.054
	(0.040)	(0.051)
Rep	0.052	−0.075
	(0.046)	(0.057)
Crime rate:Human	−0.003***	0.0001
	(0.001)	(0.001)
WFPR:Human	−0.001	−0.001
	(0.001)	(0.001)
MFPR:Human	−0.003***	−0.0002
	(0.001)	(0.001)
Crime rate:Indep/Other	−0.003***	−0.002*
	(0.001)	(0.001)
Crime rate:Rep	−0.002***	−0.001
	(0.001)	(0.001)
WFPR:Indep/Other	−0.001*	−0.001
	(0.001)	(0.001)
WFPR:Rep	−0.001**	−0.001
	(0.001)	(0.001)
MFPR:Indep/Other	−0.0003	−0.0002
	(0.001)	(0.001)
MFPR:Rep	0.002***	0.003***
	(0.001)	(0.001)
Human:Indep/Other	−0.093*	−0.039
	(0.050)	(0.055)
Human:Rep	−0.025	0.015
	(0.058)	(0.059)
Crime rate:Human:Indep/Other	0.0005	0.0004
	(0.001)	(0.001)
Crime rate:Human:Rep	−0.001	0.001
	(0.001)	(0.001)
WFPR:Human:Indep/Other	0.001	0.001
	(0.001)	(0.001)
WFPR:Human:Rep	−0.0002	0.0005
	(0.001)	(0.001)
MFPR:Human:Indep/Other	0.002**	0.001
	(0.001)	(0.001)
MFPR:Human:Rep	0.002*	0.001
	(0.001)	(0.001)
Constant	1.143***	1.140***
	(0.030)	(0.035)
Observations	64,340	31,340
R^2	0.144	0.107

Note: *p<0.1; **p<0.05; ***p<0.01. Rep = Republican, Indep = Independent.

Table S37. Heterogeneous results by political party (Republican, Democrat, or Independent/Other) for the Crime scenario. Corresponds to Hypotheses 1 and 3 in the pre-registered analysis. The dependent variable is the choice outcome.

Crime scenario

	Non-Faceoff	Faceoff	Faceoff	Faceoff
	(1)	(2)	(3)	(4)
Unfairness	−0.002***			−0.002***
	(0.0005)			(0.001)
Crime rate			−0.009***	
			(0.0005)	
WFPR			−0.005***	
			(0.0004)	
MFPR			−0.009***	
			(0.0005)	
Human	0.0001	0.041**	0.045***	0.034*
	(0.009)	(0.016)	(0.015)	(0.020)
Indep/Other	0.002	−0.023*	0.018	−0.030*
	(0.009)	(0.012)	(0.045)	(0.018)
Rep	−0.002	−0.039***	−0.108**	−0.053***
	(0.011)	(0.013)	(0.052)	(0.020)
Unfairness:Human	0.00003			0.0005
	(0.001)			(0.001)
Unfairness:Indep/Other	−0.0001			0.001
	(0.001)			(0.001)
Unfairness:Rep	0.0001			0.001
	(0.001)			(0.001)
Crime rate:Indep/Other			−0.001*	
			(0.001)	
Crime rate:Rep			−0.0003	
			(0.001)	
WFPR:Indep/Other			−0.00003	
			(0.001)	
WFPR:Rep			−0.0003	
			(0.001)	
MFPR:Indep/Other			0.0002	
			(0.001)	
MFPR:Rep			0.003***	
			(0.001)	
Human:Indep/Other	0.008	0.045*	0.035	0.045
	(0.013)	(0.023)	(0.022)	(0.030)
Human:Rep	−0.001	0.078***	0.083***	0.070**
	(0.015)	(0.026)	(0.025)	(0.033)
Unfairness:Human:Indep/Other	−0.001			−0.00000
	(0.001)			(0.001)
Unfairness:Human:Rep	0.0001			0.001
	(0.001)			(0.001)
Constant	0.533***	0.479***	1.153***	0.505***
	(0.007)	(0.008)	(0.031)	(0.012)
Observations	64,340	31,340	31,340	31,340
R^2	0.002	0.007	0.107	0.007

Note: *p<0.1; **p<0.05; ***p<0.01. Rep = Republican, Indep = Independent.

Table S38. Heterogeneous results by political party (Republican, Democrat, or Independent/Other) for the Crime scenario. Corresponds to Hypotheses 2, 4, and 5 in the pre-registered analysis. The dependent variable is the choice outcome.

Loans scenario

	Non-Faceoff	Faceoff
	(1)	(2)
Default rate	−0.036***	−0.036***
	(0.002)	(0.002)
WFPR	−0.022***	−0.022***
	(0.002)	(0.002)
MFPR	−0.037***	−0.042***
	(0.002)	(0.002)
Human	0.088***	−0.004
	(0.023)	(0.028)
Indep/Other	0.032	0.007
	(0.024)	(0.033)
Rep	0.021	−0.002
	(0.026)	(0.037)
Default rate:Human	−0.008***	0.002
	(0.003)	(0.003)
WFPR:Human	−0.005**	0.00002
	(0.002)	(0.003)
MFPR:Human	−0.005*	0.006*
	(0.003)	(0.003)
Default rate:Indep/Other	−0.006**	−0.001
	(0.003)	(0.004)
Default rate:Rep	−0.008***	−0.006
	(0.003)	(0.004)
WFPR:Indep/Other	−0.003	−0.004
	(0.003)	(0.003)
WFPR:Rep	−0.005*	−0.009**
	(0.003)	(0.004)
MFPR:Indep/Other	0.002	0.004
	(0.003)	(0.003)
MFPR:Rep	0.009***	0.013***
	(0.003)	(0.004)
Human:Indep/Other	−0.007	0.010
	(0.032)	(0.041)
Human:Rep	−0.015	0.063
	(0.035)	(0.046)
Default rate:Human:Indep/Other	−0.004	−0.001
	(0.004)	(0.004)
Default rate:Human:Rep	−0.003	−0.001
	(0.004)	(0.005)
WFPR:Human:Indep/Other	0.001	0.001
	(0.003)	(0.004)
WFPR:Human:Rep	0.002	0.005
	(0.004)	(0.005)
MFPR:Human:Indep/Other	0.004	−0.002
	(0.004)	(0.004)
MFPR:Human:Rep	0.004	−0.010**
	(0.004)	(0.005)
Constant	0.974***	0.985***
	(0.016)	(0.022)
Observations	56,480	28,320
R^2	0.146	0.125

Note: *p<0.1; **p<0.05; ***p<0.01. Rep = Republican, Indep = Independent.

Table S39. Heterogeneous results by political party (Republican, Democrat, or Independent/Other) for the Loans scenario. Corresponds to Hypotheses 1 and 3 in the pre-registered analysis. The dependent variable is the choice outcome.

Loans scenario

	Non-Faceoff	Faceoff	Faceoff	Faceoff
	(1)	(2)	(3)	(4)
Unfairness	−0.012***			−0.004
	(0.002)			(0.003)
Default rate			−0.036***	
			(0.002)	
WFPR			−0.022***	
			(0.002)	
MFPR			−0.039***	
			(0.002)	
Human	−0.011	0.038**	0.033**	0.041**
	(0.010)	(0.015)	(0.014)	(0.020)
Indep/Other	−0.008	0.005	0.011	0.022
	(0.010)	(0.011)	(0.028)	(0.017)
Rep	−0.017	−0.018	0.014	−0.009
	(0.011)	(0.013)	(0.032)	(0.020)
Unfairness:Human	0.003			−0.001
	(0.003)			(0.004)
Unfairness:Indep/Other	0.002			−0.005
	(0.003)			(0.004)
Unfairness:Rep	0.005*			−0.003
	(0.003)			(0.005)
Default rate:Indep/Other			−0.002	
			(0.003)	
Default rate:Rep			−0.006**	
			(0.003)	
WFPR:Indep/Other			−0.003	
			(0.003)	
WFPR:Rep			−0.007**	
			(0.003)	
MFPR:Indep/Other			0.003	
			(0.003)	
MFPR:Rep			0.008***	
			(0.003)	
Human:Indep/Other	0.012	−0.011	0.002	−0.017
	(0.014)	(0.022)	(0.020)	(0.029)
Human:Rep	−0.002	0.036	0.031	0.021
	(0.016)	(0.026)	(0.024)	(0.033)
Unfairness:Human:Indep/Other	−0.004			0.002
	(0.004)			(0.006)
Unfairness:Human:Rep	0.0002			0.005
	(0.004)			(0.006)
Constant	0.542***	0.481***	0.967***	0.496***
	(0.007)	(0.007)	(0.019)	(0.012)
Observations	56,480	28,320	28,320	28,320
R^2	0.002	0.002	0.125	0.003

Note: *p<0.1; **p<0.05; ***p<0.01. Rep = Republican, Indep = Independent.

Table S40. Heterogeneous results by political party (Republican, Democrat, or Independent/Other) for the Loans scenario. Corresponds to Hypotheses 2, 4, and 5 in the pre-registered analysis. The dependent variable is the choice outcome.

Crime scenario

	Non-Faceoff	Non-Faceoff	Faceoff	Faceoff	Faceoff	Faceoff
	(1)	(2)	(3)	(4)	(5)	(6)
Crime rate	−0.010***			−0.009***	−0.009***	
	(0.0004)			(0.0004)	(0.0005)	
WFPR	−0.006***			−0.005***	−0.005***	
	(0.0003)			(0.0004)	(0.0005)	
MFPR	−0.007***			−0.008***	−0.008***	
	(0.0004)			(0.0004)	(0.0005)	
Unfairness		−0.003***				−0.002***
		(0.0004)				(0.001)
Human	0.163***	−0.001	0.086***	0.088***	0.060**	0.072***
	(0.027)	(0.007)	(0.013)	(0.012)	(0.029)	(0.016)
College	0.040	−0.002	0.014	0.079**	0.067	0.002
	(0.037)	(0.009)	(0.011)	(0.040)	(0.045)	(0.016)
Crime rate:Human	−0.003***				0.001	
	(0.001)				(0.001)	
WFPR:Human	−0.0004				0.0002	
	(0.0004)				(0.001)	
MFPR:Human	−0.002***				0.0002	
	(0.0005)				(0.001)	
Crime rate:College	−0.002***			−0.001**	−0.001	
	(0.001)			(0.001)	(0.001)	
WFPR:College	0.001			−0.0002	0.0003	
	(0.001)			(0.001)	(0.001)	
MFPR:College	−0.0001			−0.0005	−0.001	
	(0.001)			(0.001)	(0.001)	
Unfairness:Human		0.0001				0.001
		(0.001)				(0.001)
Unfairness:College		0.0002				0.001
		(0.001)				(0.001)
Human:College	0.013	0.011	−0.027	−0.028	−0.003	−0.014
	(0.046)	(0.012)	(0.021)	(0.020)	(0.049)	(0.027)
Crime rate:Human:College	0.001				−0.0002	
	(0.001)				(0.001)	
WFPR:Human:College	−0.001				−0.001	
	(0.001)				(0.001)	
MFPR:Human:College	−0.0004				0.0004	
	(0.001)				(0.001)	
Unfairness:Human:College		−0.001				−0.001
		(0.001)				(0.001)
Constant	1.187***	0.534***	0.457***	1.102***	1.116***	0.481***
	(0.022)	(0.005)	(0.006)	(0.025)	(0.029)	(0.010)
Observations	64,340	64,340	31,340	31,340	31,340	31,340
R^2	0.142	0.002	0.006	0.105	0.105	0.007

Note: *p<0.1; **p<0.05; ***p<0.01.

Table S41. Heterogeneous results by education level for the Crime scenario. Corresponds to Hypotheses 1-5 in the pre-registered analysis. The dependent variable is the choice outcome.

Loans scenario

	Non-Faceoff	Non-Faceoff	Faceoff	Faceoff	Faceoff	Faceoff
	(1)	(2)	(3)	(4)	(5)	(6)
Default rate	−0.037***			−0.034***	−0.034***	
	(0.002)			(0.002)	(0.002)	
WFPR	−0.026***			−0.024***	−0.024***	
	(0.001)			(0.001)	(0.002)	
MFPR	−0.032***			−0.034***	−0.036***	
	(0.001)			(0.001)	(0.002)	
Unfairness		−0.010***				−0.008***
		(0.002)				(0.002)
Human	0.097***	−0.002	0.049***	0.048***	0.032	0.039**
	(0.017)	(0.008)	(0.012)	(0.011)	(0.023)	(0.016)
College	0.052**	−0.0005	0.009	0.108***	0.123***	−0.004
	(0.021)	(0.009)	(0.010)	(0.024)	(0.028)	(0.016)
Default rate:Human	−0.010***				0.001	
	(0.002)				(0.002)	
WFPR:Human	−0.004*				−0.0003	
	(0.002)				(0.002)	
MFPR:Human	−0.005***				0.003	
	(0.002)				(0.002)	
Deafult rate:College	−0.009***			−0.012***	−0.012***	
	(0.003)			(0.003)	(0.003)	
WFPR:College	0.006**			−0.003	−0.006*	
	(0.002)			(0.002)	(0.003)	
MFPR:College	−0.007***			−0.005**	−0.005*	
	(0.002)			(0.002)	(0.003)	
Unfairness:Human		0.001				0.003
		(0.002)				(0.003)
Unfairness:College		0.0003				0.004
		(0.003)				(0.004)
Human:College	−0.040	−0.015	−0.018	−0.017	−0.047	0.003
	(0.028)	(0.013)	(0.020)	(0.019)	(0.036)	(0.027)
Default rate:Human:College	−0.001				0.0004	
	(0.004)				(0.004)	
WFPR:Human:College	−0.002				0.005	
	(0.003)				(0.004)	
MFPR:Human:College	0.009***				0.0002	
	(0.003)				(0.004)	
Unfairness:Human:College		0.004				−0.006
		(0.004)				(0.005)
Constant	0.973***	0.535***	0.475***	0.937***	0.945***	0.503***
	(0.013)	(0.005)	(0.006)	(0.016)	(0.019)	(0.009)
Observations	56,480	56,480	28,320	28,320	28,320	28,320
R^2	0.146	0.002	0.002	0.126	0.126	0.003

Note: *p<0.1; **p<0.05; ***p<0.01.

Table S42. Heterogeneous results by education level for the Loans scenario. Corresponds to Hypotheses 1-5 in the pre-registered analysis. The dependent variable is the choice outcome.

Crime scenario

	Non-Faceoff	Non-Faceoff	Faceoff	Faceoff	Faceoff	Faceoff
	(1)	(2)	(3)	(4)	(5)	(6)
Crime rate	−0.011***			−0.010***	−0.010***	
	(0.0004)			(0.0004)	(0.0005)	
WFPR	−0.005***			−0.005***	−0.005***	
	(0.0003)			(0.0003)	(0.0004)	
MFPR	−0.007***			−0.008***	−0.008***	
	(0.0003)			(0.0003)	(0.0004)	
Unfairness		−0.003***				−0.002***
		(0.0004)				(0.0005)
Human	0.169***	0.001	0.056***	0.057***	0.064**	0.043***
	(0.026)	(0.007)	(0.012)	(0.011)	(0.028)	(0.015)
LowAI	0.031	−0.016*	−0.039***	−0.012	0.035	−0.048***
	(0.040)	(0.009)	(0.011)	(0.045)	(0.048)	(0.017)
Crime rate:Human	−0.003***				0.0001	
	(0.0005)				(0.001)	
WFPR:Human	−0.001				−0.001	
	(0.0004)				(0.001)	
MFPR:Human	−0.002***				0.0002	
	(0.0004)				(0.001)	
Crime rate:LowAI	−0.0004			0.0001	−0.001	
	(0.001)			(0.001)	(0.001)	
WFPR:LowAI	−0.001			−0.0004	−0.001	
	(0.001)			(0.001)	(0.001)	
MFPR:LowAI	0.0002			−0.001	−0.001	
	(0.001)			(0.001)	(0.001)	
Unfairness:Human		−0.00004				0.001
		(0.0005)				(0.001)
Unfairness:LowAI		0.001*				0.001
		(0.001)				(0.001)
Human:LowAI	−0.004	0.007	0.077***	0.077***	−0.017	0.091***
	(0.050)	(0.013)	(0.023)	(0.022)	(0.052)	(0.029)
Crime rate:Human:LowAI	−0.0003				0.002	
	(0.001)				(0.001)	
WFPR:Human:LowAI	−0.0001				0.001	
	(0.001)				(0.001)	
MFPR:Human:LowAI	0.0005				0.0002	
	(0.001)				(0.001)	
Unfairness:Human:LowAI		−0.001				−0.001
		(0.001)				(0.001)
Constant	1.193***	0.537***	0.472***	1.134***	1.131***	0.495***
	(0.020)	(0.005)	(0.006)	(0.023)	(0.026)	(0.009)
Observations	64,340	64,340	31,340	31,340	31,340	31,340
R^2	0.142	0.002	0.007	0.106	0.106	0.008

Note: *p<0.1; **p<0.05; ***p<0.01.

Table S43. Heterogeneous results by AI pessimism for the Crime scenario. Corresponds to Hypotheses 1-5 in the pre-registered analysis. "LowAI" indicates users who are pessimistic (below the median) regarding the use of AI in society. The dependent variable is the choice outcome.

Loans scenario

	Non-Faceoff	Non-Faceoff	Faceoff	Faceoff	Faceoff	Faceoff
	(1)	(2)	(3)	(4)	(5)	(6)
Default rate	−0.040***			−0.037***	−0.038***	
	(0.001)			(0.001)	(0.002)	
WFPR	−0.024***			−0.023***	−0.024***	
	(0.001)			(0.001)	(0.002)	
MFPR	−0.034***			−0.035***	−0.037***	
	(0.001)			(0.001)	(0.002)	
Unfairness		−0.011***				−0.005**
		(0.001)				(0.002)
Human	0.071***	−0.012	0.032***	0.032***	−0.013	0.034**
	(0.016)	(0.007)	(0.011)	(0.010)	(0.021)	(0.014)
LowAI	0.007	−0.007	−0.023**	0.058**	0.023	0.003
	(0.023)	(0.010)	(0.011)	(0.027)	(0.032)	(0.018)
Default rate:Human	−0.009***				0.002	
	(0.002)				(0.002)	
WFPR:Human	−0.004**				0.003	
	(0.002)				(0.002)	
MFPR:Human	−0.001				0.004*	
	(0.002)				(0.002)	
Default rate:LowAI	0.002			−0.003	−0.001	
	(0.003)			(0.003)	(0.004)	
WFPR:LowAI	−0.002			−0.008***	−0.006*	
	(0.002)			(0.002)	(0.003)	
MFPR:LowAI	−0.001			−0.004	−0.001	
	(0.003)			(0.003)	(0.003)	
Unfairness:Human		0.003*				−0.001
		(0.002)				(0.003)
Unfairness:LowAI		0.002				−0.007*
		(0.003)				(0.004)
Human:LowAI	0.049	0.017	0.045**	0.043**	0.114***	0.025
	(0.030)	(0.014)	(0.022)	(0.020)	(0.039)	(0.030)
Default rate:Human:LowAI	−0.002				−0.004	
	(0.004)				(0.004)	
WFPR:Human:LowAI	−0.003				−0.004	
	(0.003)				(0.004)	
MFPR:Human:LowAI	−0.005				−0.005	
	(0.004)				(0.004)	
Unfairness:Human:LowAI		−0.005				0.006
		(0.004)				(0.006)
Constant	0.989***	0.537***	0.484***	0.961***	0.983***	0.501***
	(0.012)	(0.005)	(0.006)	(0.014)	(0.017)	(0.009)
Observations	56,480	56,480	28,320	28,320	28,320	28,320
R^2	0.145	0.002	0.002	0.125	0.125	0.003

Note: *p<0.1; **p<0.05; ***p<0.01.

Table S44. Heterogeneous results by AI pessimism for the Loans scenario. Corresponds to Hypotheses 1-5 in the pre-registered analysis. "LowAI" indicates users who are pessimistic (below the median) regarding the use of AI in society. The dependent variable is the choice outcome.

Additional Figures

	Performance of ALGORITHM A	Performance of ALGORITHM B
DEFENDANT CRIME RATE: Percent of defendants who commit crime after being mistakenly released	13%	32%
WHITE FALSE POSITIVE RATE: Percent of White defendants who were low risk but mistakenly held in jail	49%	11%
MINORITY FALSE POSITIVE RATE: Percent of Minority defendants who were low risk but mistakenly held in jail	25%	33%

Fig. S1. Example of conjoint profile pair: Crime scenario, Algorithms condition

	Performance of MANAGER A	Performance of MANAGER B
LOAN DEFAULT RATE: Percent of applicants who default on their loans	2.5%	9.0%
WHITE FALSE POSITIVE RATE: Percent of White applicants who were low risk but mistakenly denied	4.0%	2.0%
MINORITY FALSE POSITIVE RATE: Percent of Minority applicants who were low risk but mistakenly denied	6.0%	3.5%

Fig. S2. Example of conjoint profile pair: Loans scenario, Humans condition

	Performance of ALGORITHM	Performance of MANAGER
**LOAN DEFAULT RATE: Percent of applicants who default on their loans**	2.5%	9.0%
**WHITE FALSE POSITIVE RATE: Percent of White applicants who were low risk but mistakenly denied**	0.5%	7.0%
**MINORITY FALSE POSITIVE RATE: Percent of Minority applicants who were low risk but mistakenly denied**	7.0%	4.5%

Fig. S3. Example of conjoint profile pair: Loans scenario, Faceoff condition

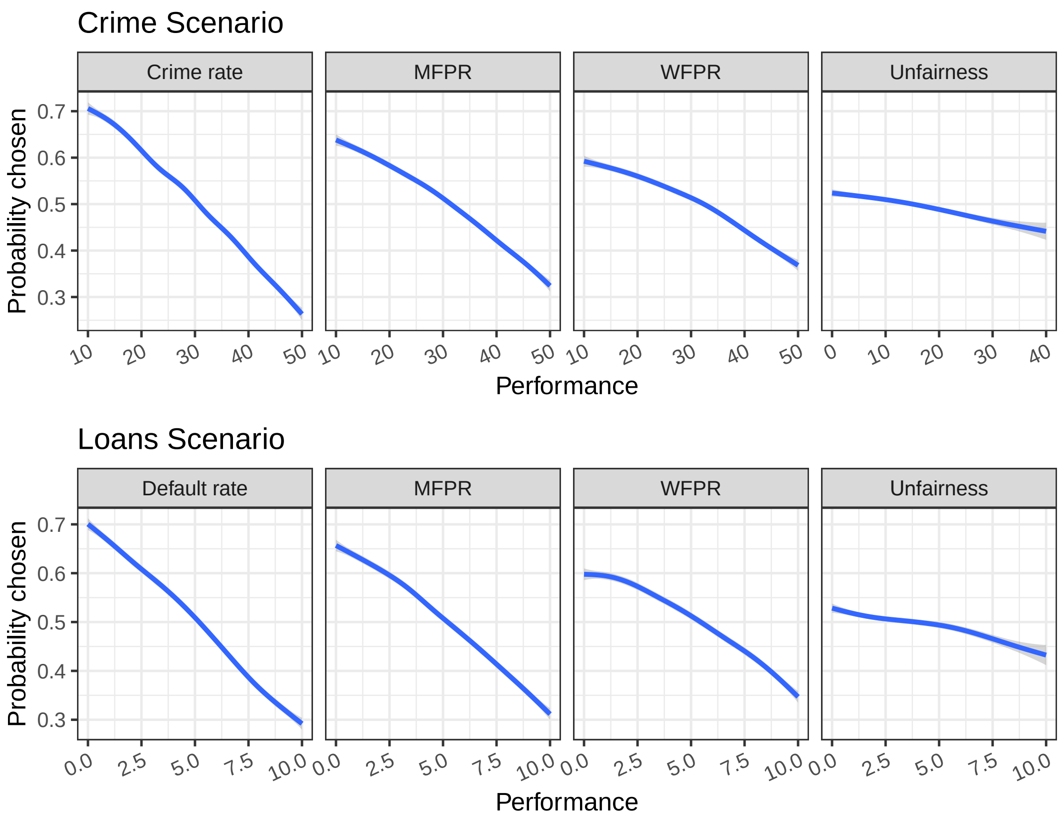

Fig. S4. LOESS-smoothed relationship between performance metrics and the probability that a given DM is chosen.

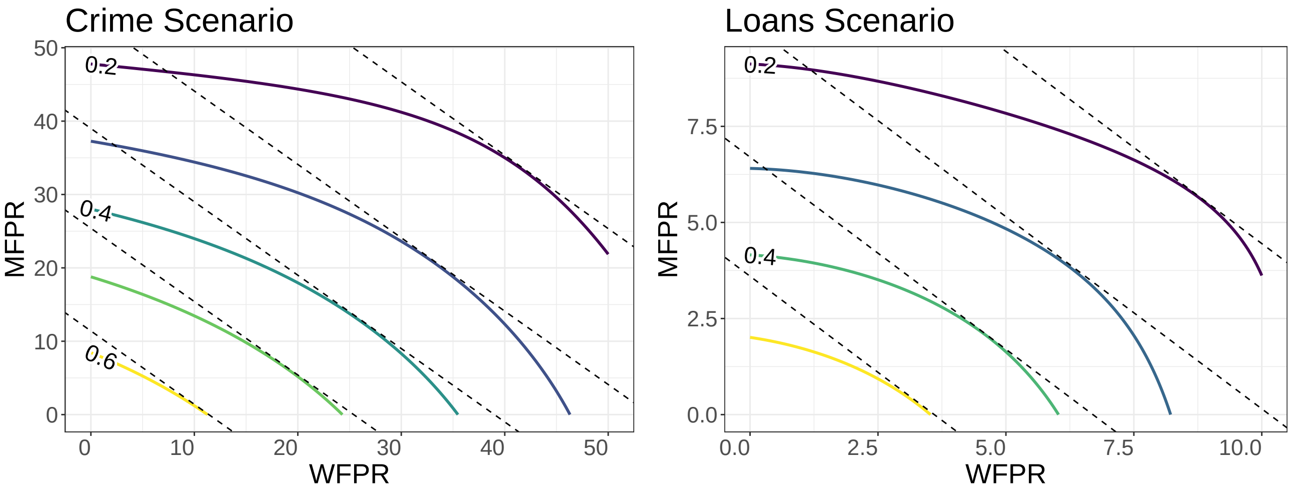

Fig. S5. Isoplot of choice probability as a function of WFPR and MFPR using all crime scenario data (left) and loan scenario data (right). The 40% isocurve, for example, corresponds to values of MFPR and WFPR such that the respondents, on average, have a 40% chance of choosing the DM. Dashed lines correspond to the tangent lines with a slope of -1. Isocurves were generated based on a ridge regression with Crime/Default rate, MFPR, and WFPR as the independent variables, with λ chosen via cross-validation. The regression included first, second, and third-order terms for each variable as well as all possible interactions. $n = 95,680$ $(4,784$ respondents each evaluating 20 DM profiles) in Crime scenario, and $n = 84,800$ $(4,240$ respondents) in Loans scenario. At the point where the tangent line touches the isocurve, respondents are indifferent between a one percentage point increase and decrease, or vice versa, in WFPR and MFPR. Interestingly, the tangent lines do not touch the isocurves at a point where MFPR is equal to WFPR, but rather intersect at a point where WFPR is higher than MFPR across all scenarios and isocurves. In line with the coefficients in Table 1, this indicates a greater relative importance placed on MFPR than WFPR in general. The shape of the isocurves illustrates a certain amount of fairness-seeking behavior. If respondents did not care about equalizing FPRs, the isocurves would be straight lines. However, this is not the case. Consider the 20% isocurve for the Crime scenario in the figure. When MFPR is high but WFPR is low, the slope of the isocurve is relatively flat, indicating that respondents are willing to substantially increase WFPR (by 15 percentage points, for example) for a small reduction (5 percentage points) in MFPR. Therefore, when the FPRs are highly unequal, and in particular when the WFPR is much lower than the MFPR, respondents exhibit more intensive fairness-seeking behavior.

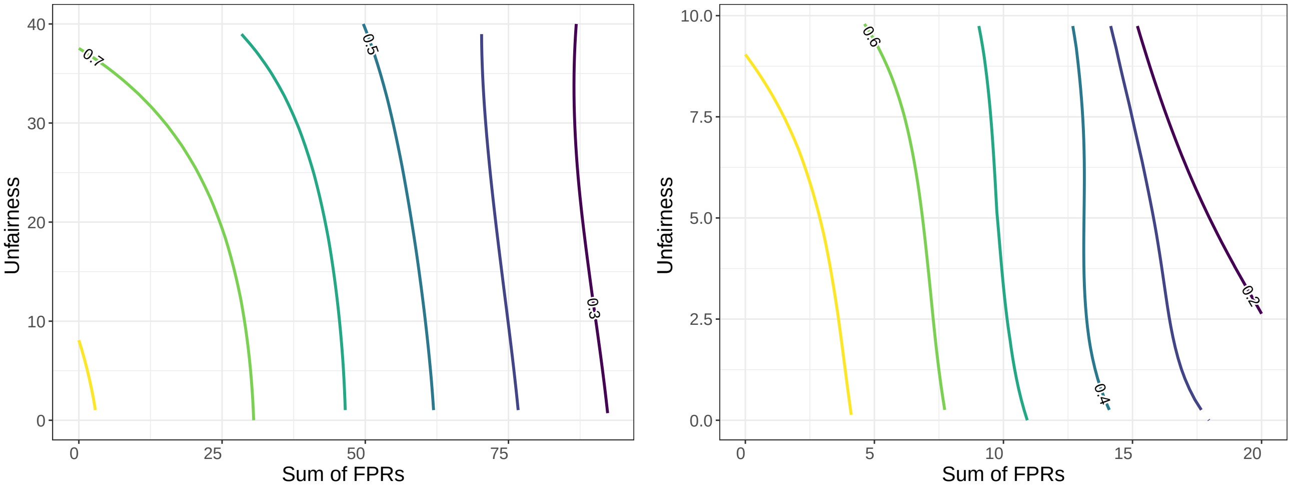

Fig. S6. Isocurves of fairness versus the sum of false positive rates (MFPR + WFPR) for the Crime scenario (left) and Loan scenario (right). Isocurves were generated based on a ridge regression with Fairness and MFPR+WFPR as the independent variables, with λ chosen via cross-validation. The regression included first, second, and third-order terms for each variable as well as all possible interactions.

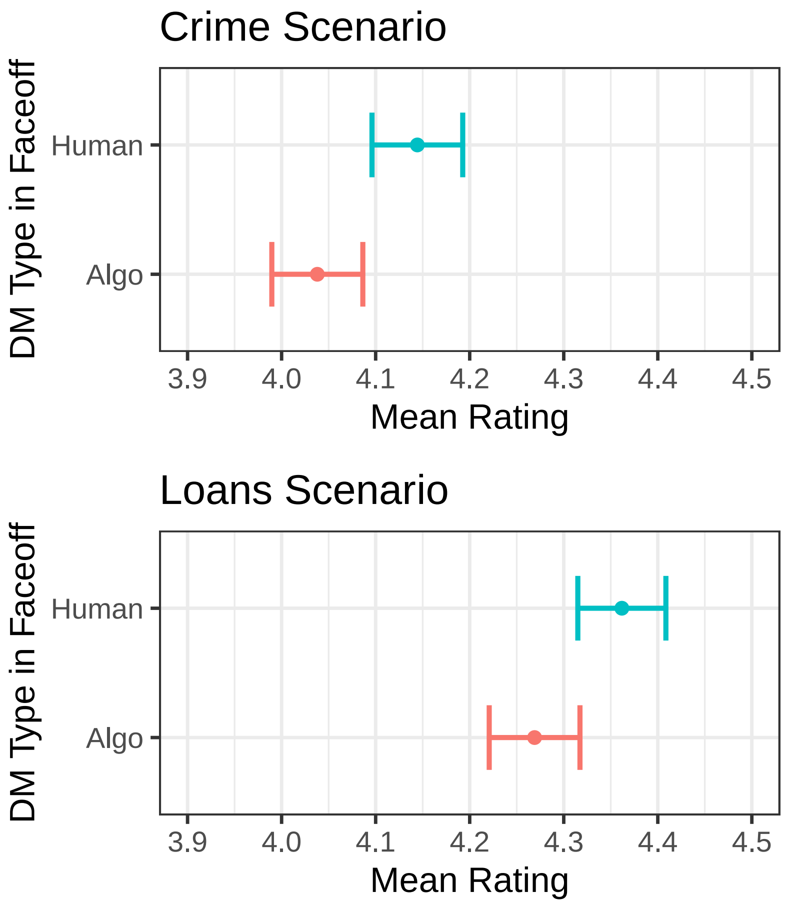

Fig. S7. Average rating of DM type in the Faceoff condition for Crime scenario (left) and Loan scenario (right). $n = 31,340$ $(1,567$ respondents each evaluating 20 DM profiles) in Crime scenario, and $n = 28,320$ $(1,416$ respondents) in Loans scenario. 95% confidence intervals displayed. Underlying estimates can be found in Table S6.

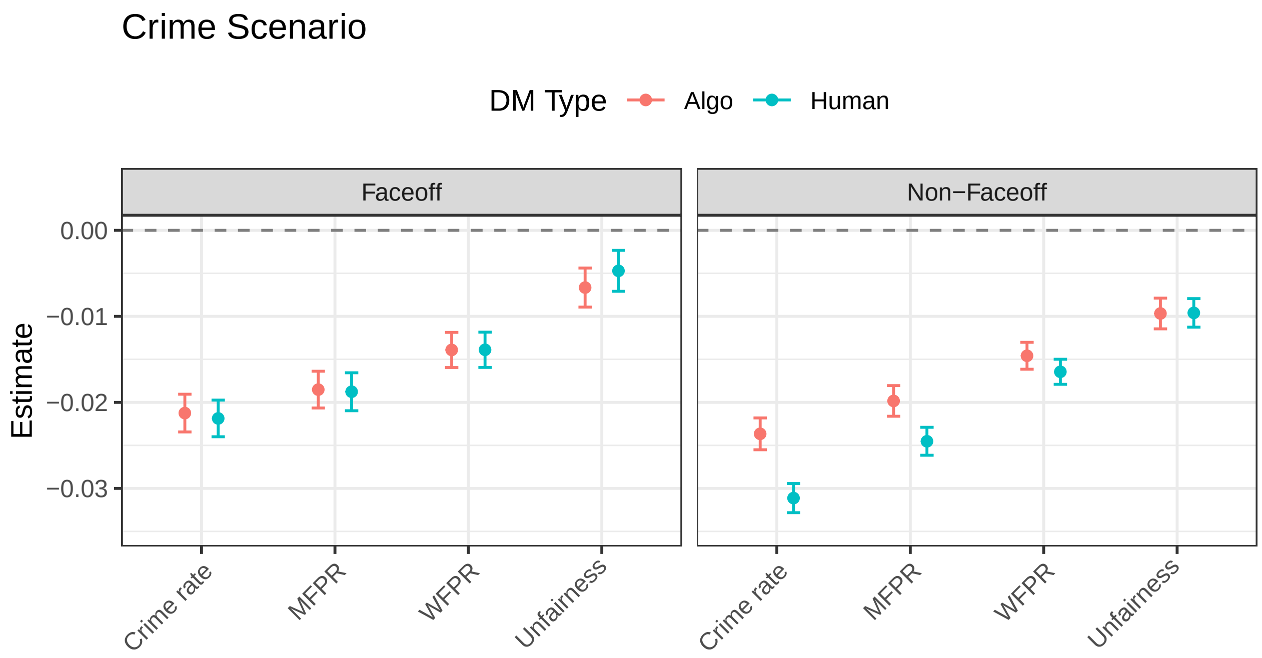

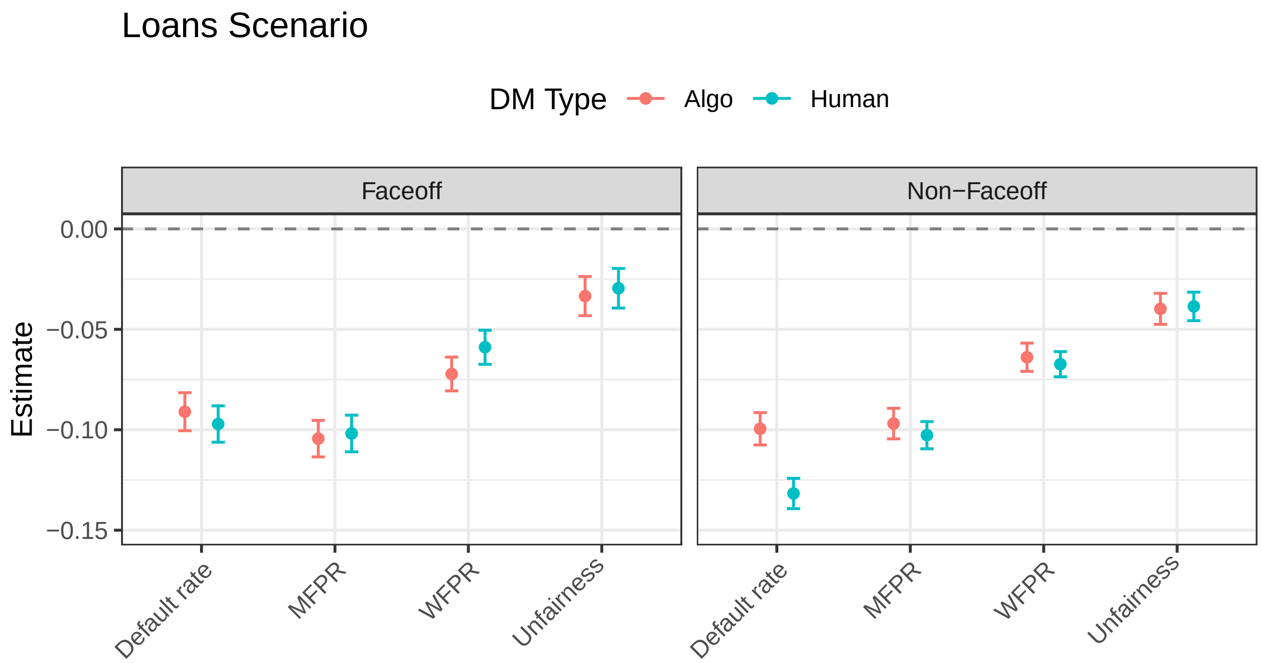

Fig. S8. Linear regression coefficient on the four performance metrics when predicting the respondents' rating.

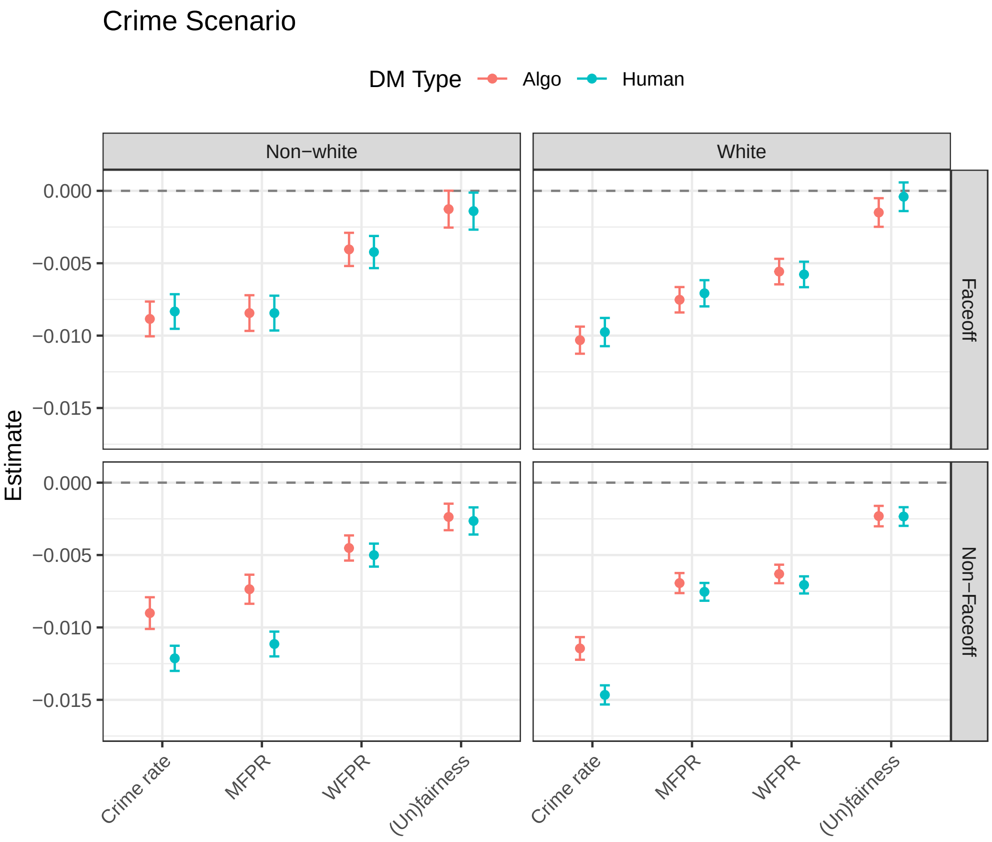

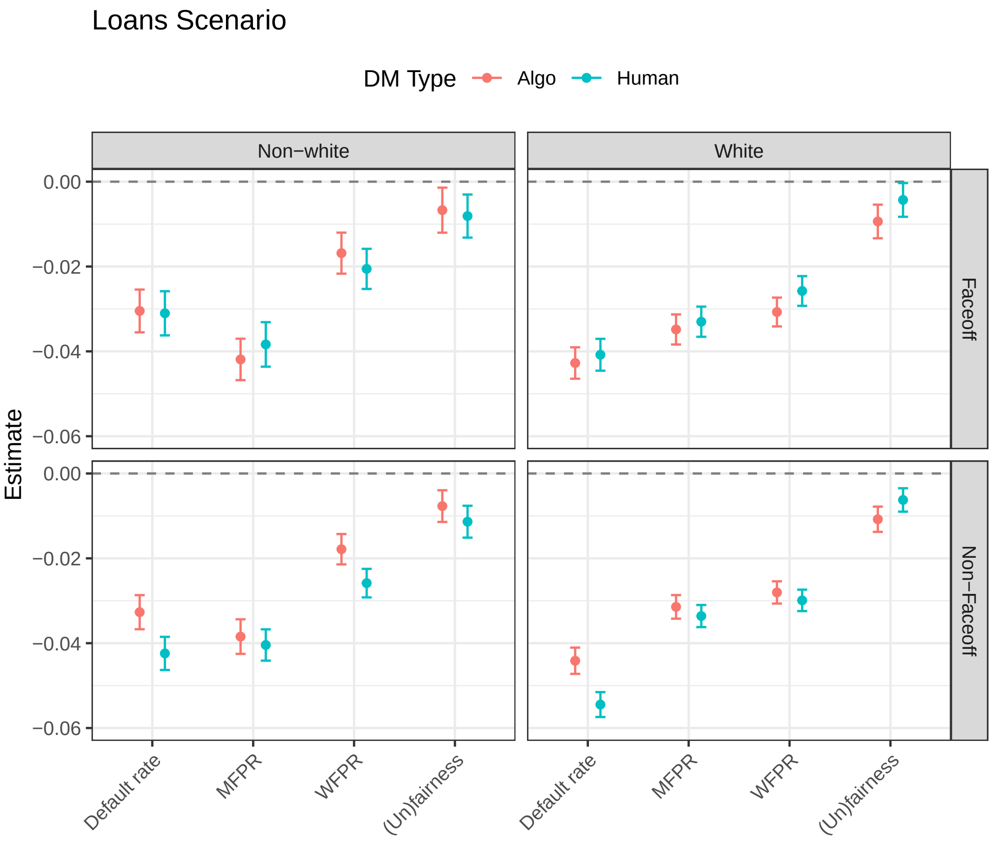

Fig. S9. Linear regression coefficient on the four performance metrics when predicting the respondents' choice, by respondents' race (non-white vs. white).

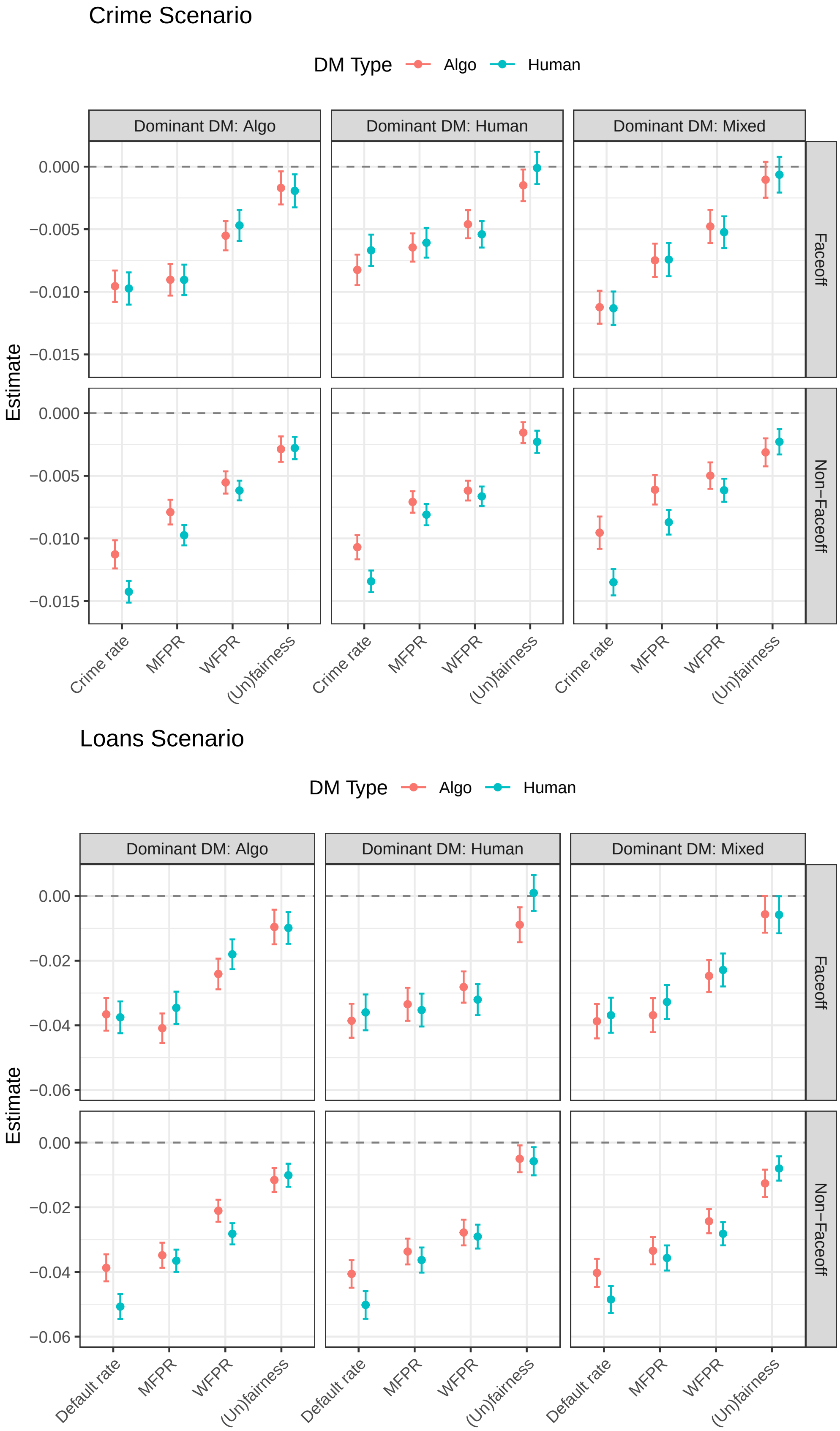

Fig. S10. Linear regression coefficient on the four performance metrics when predicting the respondents' choice, by respondents' beliefs about which DM type displays dominant performance in the real world.

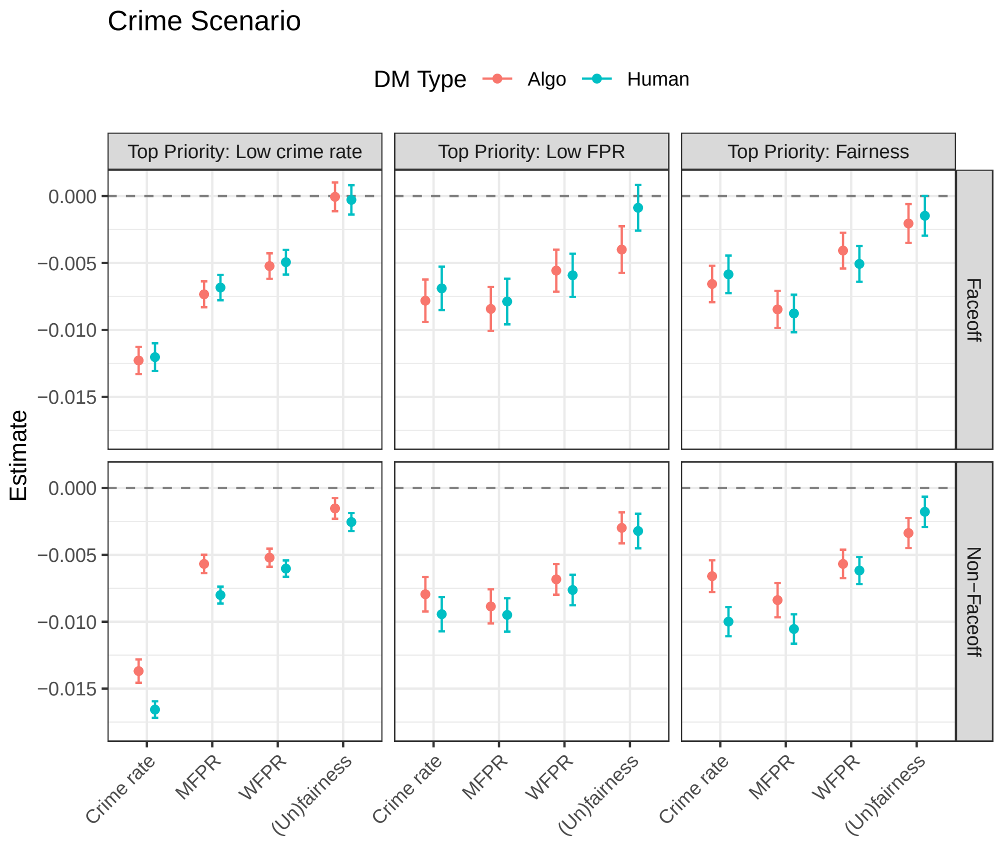

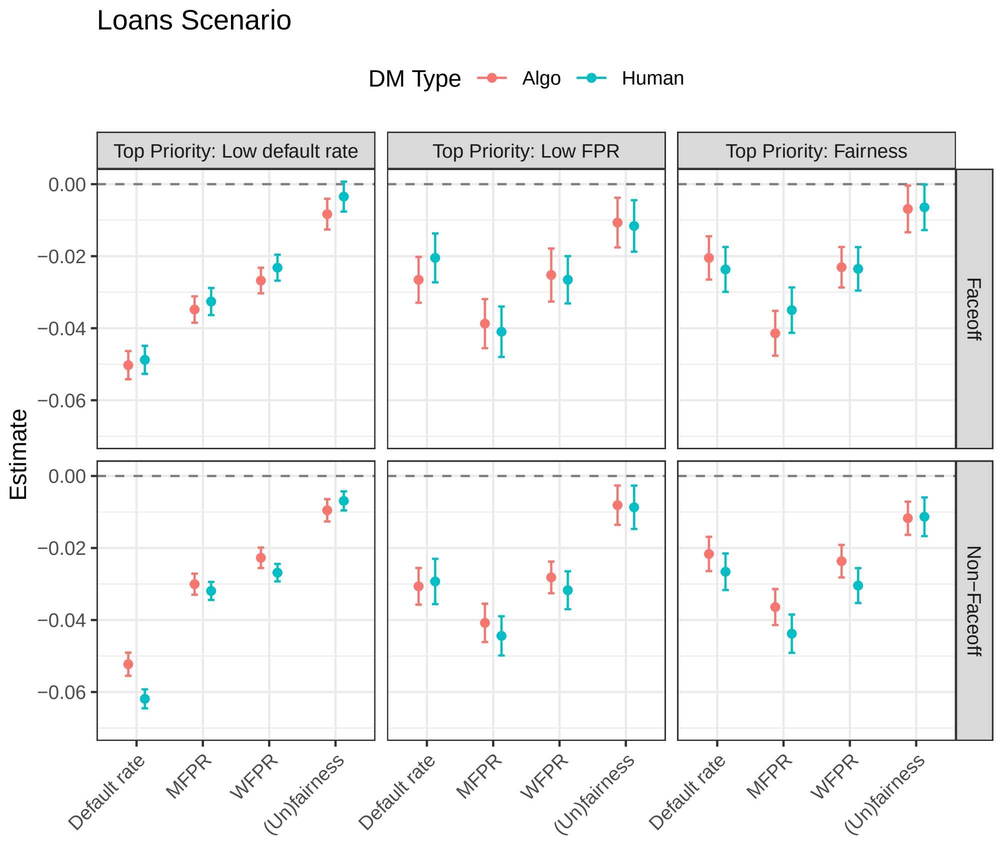

Fig. S11. Linear regression coefficient on the four performance metrics when predicting the respondents' choice, by respondents' self-reported performance priorities.

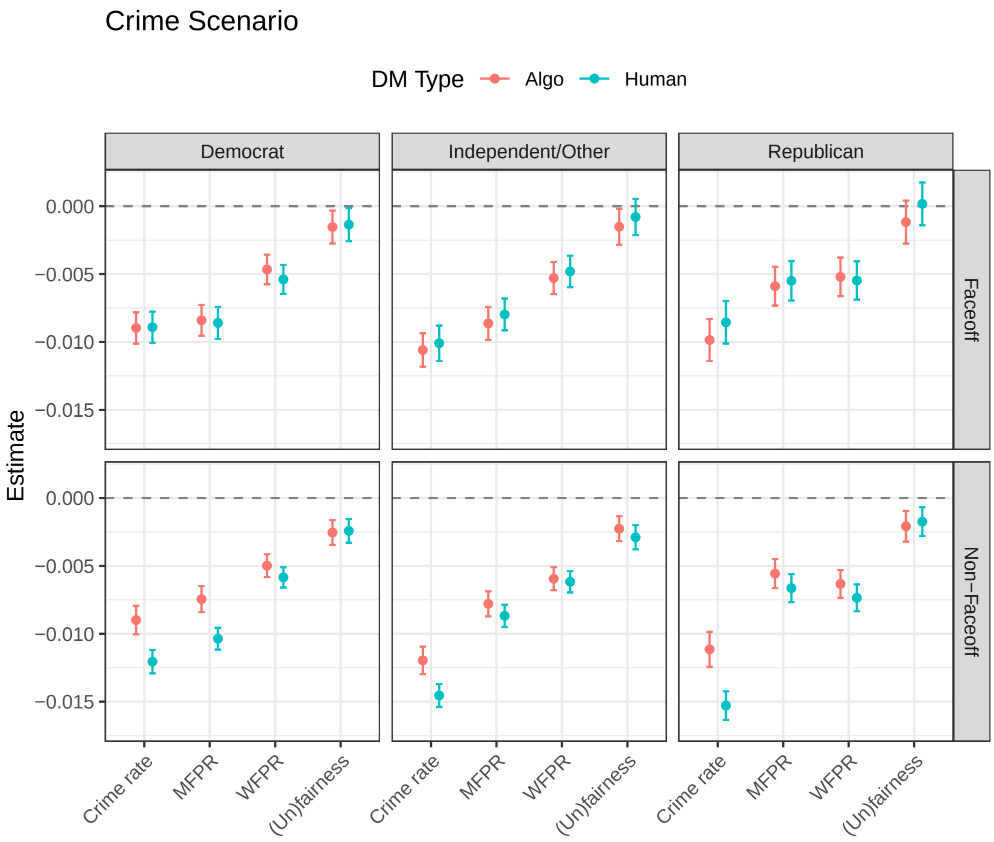

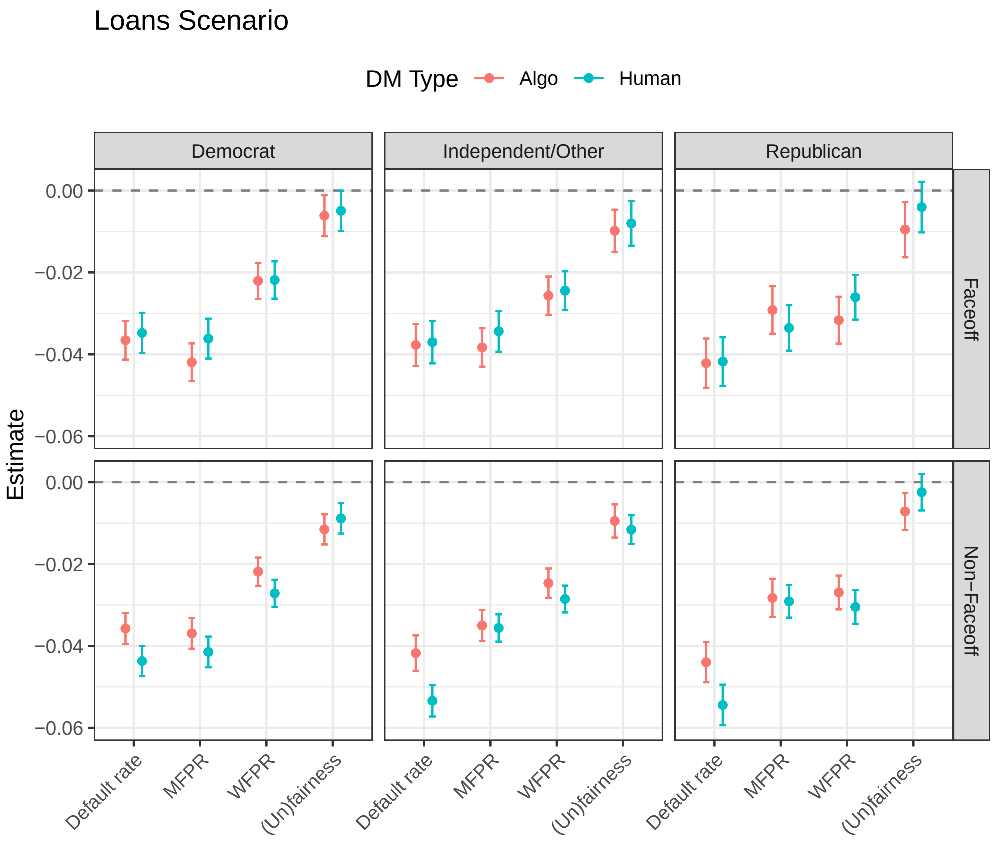

Fig. S12. Linear regression coefficient on the four performance metrics when predicting the respondents' choice, by respondents' political party identification.

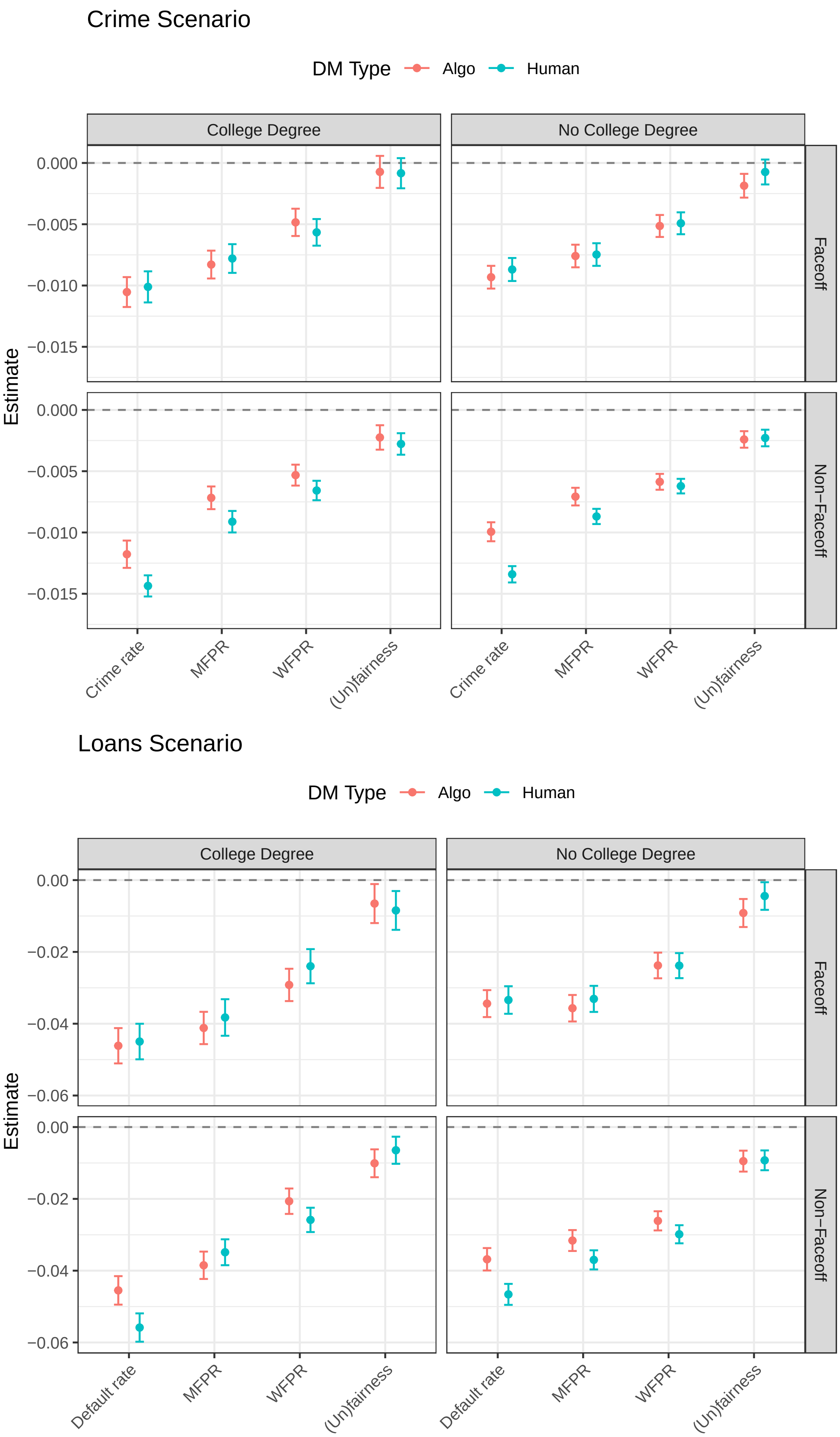

Fig. S13. Linear regression coefficient on the four performance metrics when predicting the respondents' choice, by respondents' education (college degree vs. no college degree).

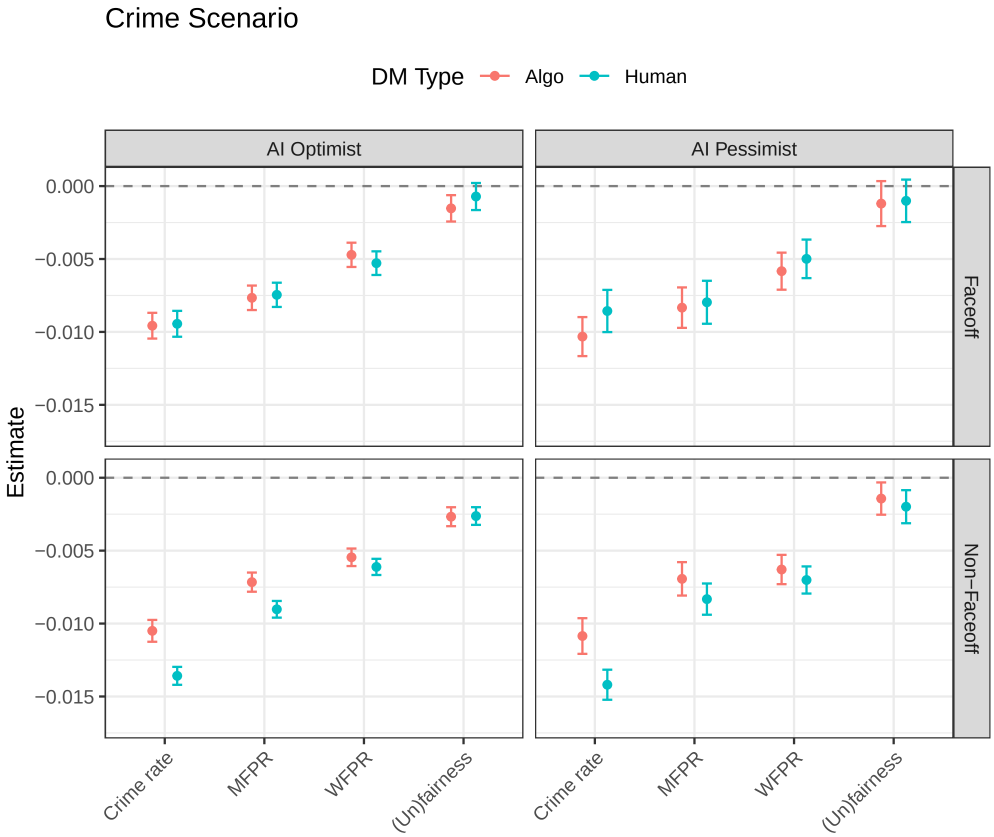

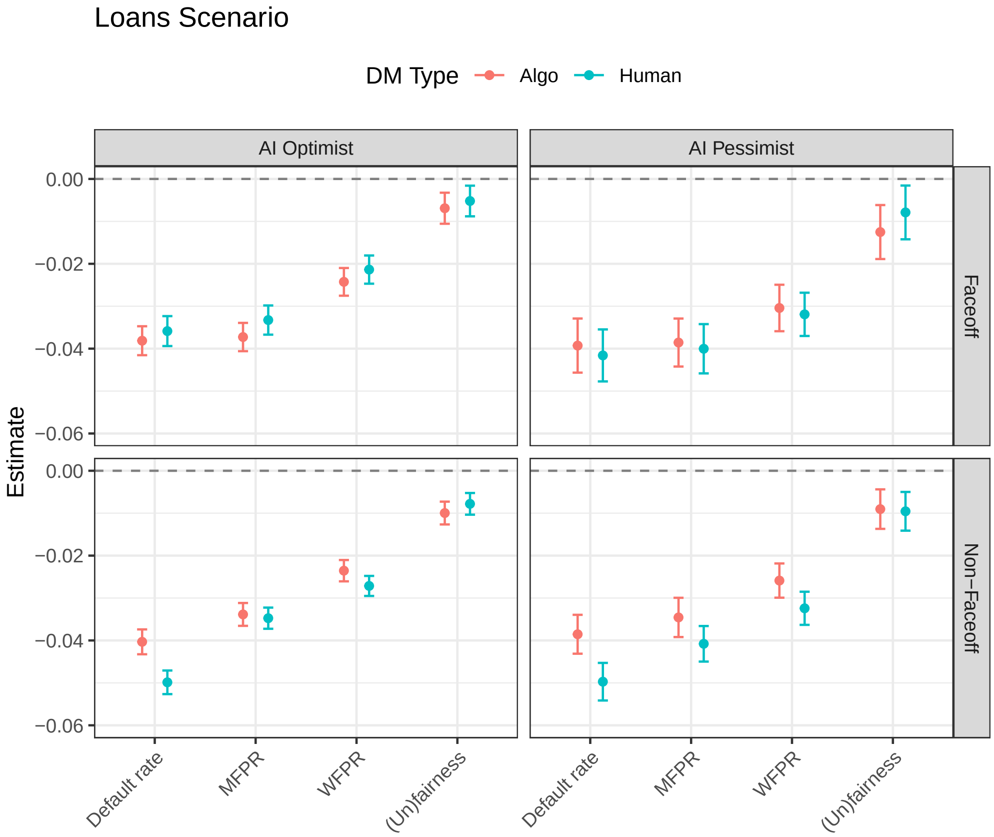

Fig. S14. Linear regression coefficient on the four performance metrics when predicting the respondents' choice, by respondents' self-reported attitudes toward AI, specifically whether respondent is an AI pessimist (below median value on AI attitudes index) or optimist (equal to or above median value).

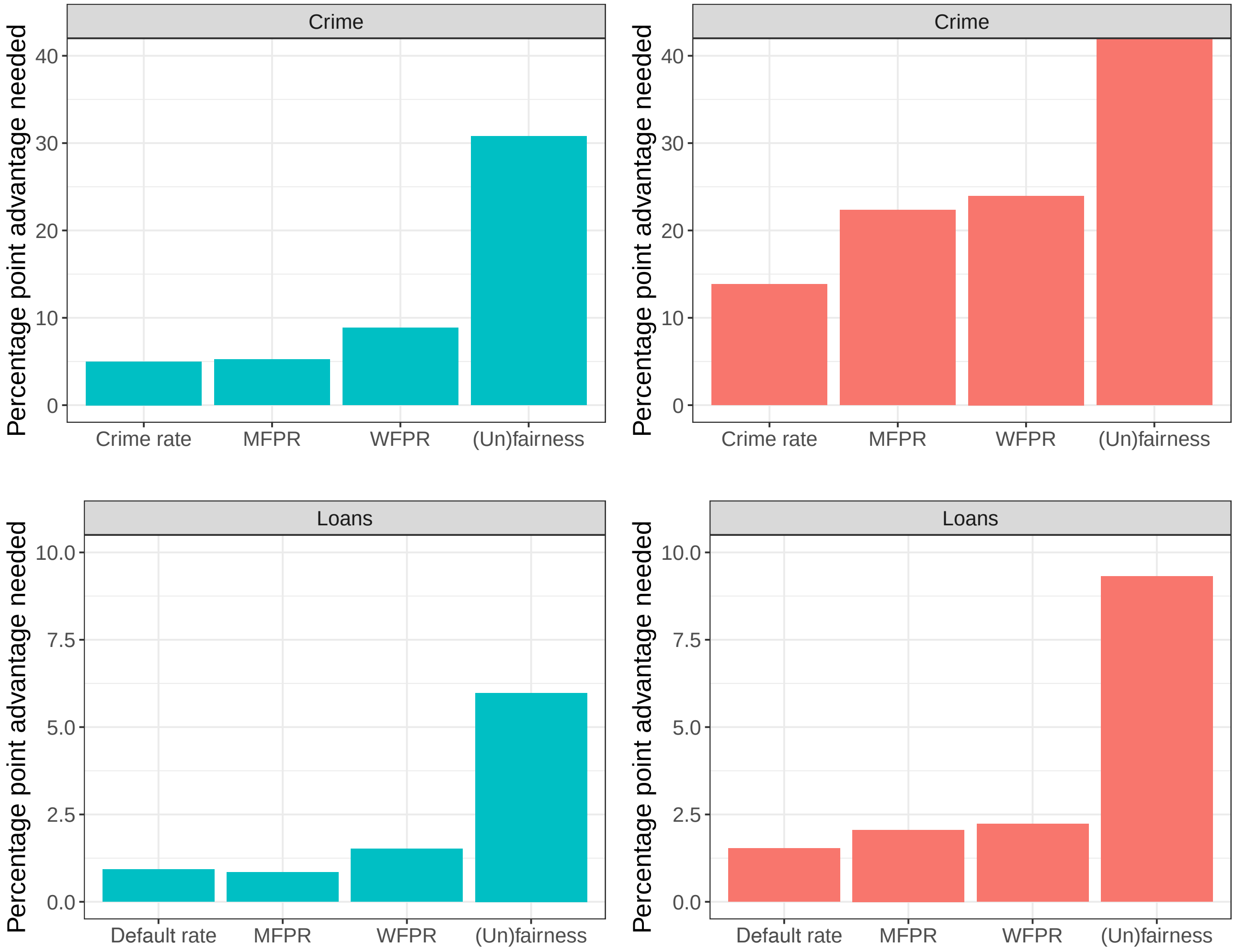

Fig. S15. Average percentage point decrease in each metric needed for algorithmic DM to have the same probability as being chosen as the human DM, using Faceoff data. The left column (in blue) shows results for Democrats and the right column (in red) shows results for Republicans.
